# Reproducibility and Respiratory Function Correlates of Exhaled Breath Fingerprint in Chronic Obstructive Pulmonary Disease

**DOI:** 10.1371/journal.pone.0045396

**Published:** 2012-10-15

**Authors:** Raffaele Antonelli Incalzi, Giorgio Pennazza, Simone Scarlata, Marco Santonico, Massimo Petriaggi, Domenica Chiurco, Claudio Pedone, Arnaldo, D'Amico

**Affiliations:** 1 Chair of Geriatrics, Unit of Respiratory Pathophysiology, Campus Bio-Medico University, Rome, Italy; 2 San Raffaele-Cittadella della Carità Foundation, Taranto, Italy; 3 Center for Integrated Research – CIR, Unit of Electronics for Sensor Systems, Campus Bio-Medico University, Rome, Italy; 4 Department of Electronic Engineering, University of Rome “Tor Vergata”, Rome, Italy; Clinica Universidad de Navarra, Spain

## Abstract

**Background:**

The electronic nose (e nose) provides distinctive breath fingerprints for selected respiratory diseases. Both reproducibility and respiratory function correlates of breath fingerprint are poorly known.

**Objectives:**

To measure reproducibility of breath fingerprints and to assess their correlates among respiratory function indexes in elderly healthy and COPD subjects.

**Method:**

25 subjects (5 COPD patients for each GOLD stage and 5 healthy controls) over 65 years underwent e-nose study through a seven sensor system and respiratory function tests at times 0, 7, and 15 days. Reproducibility of the e nose pattern was computed. The correlation between volatile organic compound (VOC) pattern and respiratory function/clinical parameters was assessed by the Spearman's rho.

**Measurements and Main Results:**

VOC patterns were highly reproducible within healthy and GOLD 4 COPD subjects, less among GOLD 1–3 patients.VOC patterns significantly correlated with expiratory flows (Spearman's rho ranging from 0.36 for MEF25% and sensor Co-Buti-TPP, to 0.81 for FEV1% and sensor Cu-Buti-TPP p<0.001)), but not with residual volume and total lung capacity.

**Conclusions:**

VOC patterns strictly correlated with expiratory flows. Thus, e nose might conveniently be used to assess COPD severity and, likely, to study phenotypic variability. However, the suboptimal reproducibility within GOLD 1–3 patients should stimulate further research to identify more reproducible breath print patterns.

## Introduction

The electronic nose (e-nose) technology has been used to typify exhaled breath for research purposes. This technique provides a sort of finger print of exhaled breath by detecting different volatile organic compounds (VOCs) through multiple sensors. Though the VOCs corresponding to individual components of exhaled breath profiles remain largely unknown, it is of interest that the resulting profile has been shown to distinguish cancer from non cancer respiratory patients as if lung cancer were associated with the release of distinctive VOCs by malignant cells and cancer-induced inflammation [1], [2]. The e-nose has also been able to separate asthmatics from healthy controls [3] and from COPD patients, based on well distinguished exhaled breath patterns, likely reflecting the well known differences in pathogenetic mechanisms of asthma and COPD [4]. These findings suggest that exhaled breath qualifies as a sort of “breath print” of selected diseases, and, thus, might be useful for diagnostic purposes as well as to monitor the response to therapy.

The use of the e nose in COPD population seems of special interest for many reasons. First, COPD is a heterogeneous disease encompassing a variety of phenotypic expressions which are far from being univocally defined [5]. Second, bronchial inflammation and a pro-oxidative status, both common in COPD patients, are expected to impact the VOCs patterns [6]. Third, changes in VOCs pattern might be a clue to the early diagnosis of COPD exacerbation, a frequently unrecognized condition [7]. Fourth, elderly people are frequently unable to satisfactorily perform spirometry [8]; this makes alternative diagnostic methods highly desirable, mainly because of the age-related dramatic increase in prevalence of COPD [9]. Finally, non invasiveness and easiness are unique features making e-nose worthy of special interest in frail and frequently disabled patients.

Preliminary to the use of e-nose as a research and, hopefully, a clinical diagnostic tool in COPD populations is the knowledge of its reproducibility as well as of its main correlates among respiratory function parameters. As far as we know, repeatability (within day measurements), but not reproducibility of e-nose patterns has so far been assessed [10]. Repeatability of the electronic nose measurements was assessed in 10 healthy subjects for which a measurement was performed every day at the same time for 6 consecutive days ([11]; online supplemental data). In other studies repeatability check has been performed by the execution duplicate measurements for each patient [2]–[4], [12]. Furthermore, it is unknown to which respective extent FEV1, mid- and late-expiratory flows and indexes of gas exchange efficiency correlate with e nose derived VOCs. Clarifying this issue might help to understand the potential clinical applications of recorded VOCs. Indeed, selected respiratory function indexes such as FEV1 and FVC play a primary diagnostic and classificatory role of respiratory diseases, while others such as MEF50 or MEF75 have uncertain clinical meaning. Accordingly, defining the pattern of respiratory function correlates of VOCs and the strengths of individual correlations is expected to pave the way to the routine clinical use of VOCs. In this perspective, reproducibility and correlations are both worthy of assessment.

We designed this proof of concept study to assess reproducibility of e nose measurements in a COPD population and to verify to which extent VOCs pattern correlates with respiratory function and health status indexes of COPD severity. Finding good repeatability and identifying the main respiratory function correlates of VOCs patterns in COPD would allow test e-nose derived VOCs as biomarker of COPD and likely source of information on phenotypic variability.

## Materials and Methods

### Study participants and design

Twenty subjects with COPD (5 subject for each GOLD stage) aged 65 years and older were consecutively recruited among those attending the pulmonary medicine outpatient clinic of the University Hospital “Campus Bio-Medico” in Rome (Italy). Diagnosis of COPD was ascertained according to the American Thoracic Society/European Respiratory Society (ATS/ERS) guidelines [13] and COPD severity rated according to GOLD classification [14]. Throughout all the study COPD patients had to be in stable conditions defined as follows: usual levels of physical activity and dyspnea, rated respectively through the PASE questionnaire and the MRC score [15], [16], no change in either volume or quality of sputum, no new symptoms nor therapy modification in the month prior to the enrolment. Patients with a diagnosis of cancer or asthma, which are known to potentially affect VOC's pattern, were excluded. The diagnosis of asthma relied on criteria previously used in an elderly population (SaRA) [17].

Sample size has been calculated using the following formula [18]:
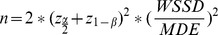
where n is the sample size; 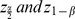 are the percentage points of the normal distribution for statistical significance level α and power 1−β, respectively; WSSD is the Within Subject Standard Deviation; MDE is the Minimum Difference Expected between the groups.

In the case of the present work, α = 0.05 and 1−β = 95% have been considered. WSSD and MDE have been calculated for each of the interested populations (Controls, Gold 1 to 4) on the basis of a previous work [19]. The final n obtained by the cited formula for each population is 5. Thus the total population consists of 25 subjects (5×5). Each subject has been longitudinally measured three times (once a week for a period of three weeks).

At the screening visit, participants underwent multidimensional assessment (clinical history, physical performance and disability, anthropometric measurements, laboratory analyses: serum multiple analysis-12, haemochrome, TSH, nutritional status, NTpro-BNP and echocardiography to rule out left ventricular dysfunction,). Comorbid conditions were identified on the basis of the patients' documentation, history, physical examination, routine blood analysis, chest x ray and ECG. Further diagnostic tests were performed, if needed. All GOLD 1–3 patients had nocturnal oximetry performed as a screening for nocturnal hypoxemia, while polisomnography was performed during regular O2 therapy in all GOLD 4 patients and only in GOLD 1–3 patients with an abnormal oximetry. In two cases, a subclinical mild Obstructive Sleep Apnea Syndrome has been diagnosed (Apnea/Hypopnea Index of 8/h and 5/h, respectively). High resolution computed tomography of the chest was performed only in patients with productive cough to rule out bronchiectasis or if chest × ray findings made necessary to exclude coexisting fibrosis. Respiratory therapy was standardized according to GOLD guidelines [13].

Five healthy subjects aged 65 and older selected out of a group attending an educational program volunteered to form the control group. They were recognized as healthy on the basis of the diagnostic work up described for COPD patients and spirometry.

Pulmonary function tests (flow-volume curves, lung volumes and carbon monoxide lung diffusion determination) and breath analysis through e nose were obtained at times 0, 7 and 15 days. Thus, both study and control subjects performed a complete set of respiratory function tests and e-nose measurements three times.

All the study participants provided written informed consent. The study protocol was approved by the local Ethical Committee (protocol number: 4711).

### Pulmonary function tests

Respiratory function tests and blood gas analysis were performed in the morning with participants fasting and smoking free for at least 12 hours [20] and twice at weekly intervals. The final dataset therefore counted 74 measurements, being a measure missed due to technical reasons. Forced expiratory volumes were measured using a water-sealed bell spirometer (Biomedin, Padua, Italy) following the acceptability and reproducibility criteria proposed by the American Thoracic Society and the European Respiratory Society (ATS/ERS) [13] after a 24 hours wash out from inhaled therapy. The maneuver was repeated after inhalation of salbutamol and post-bronchodilator data were used to characterize COPD patients [21]. Total Lung Capacity (TLC) and Residual Volume (RV) were obtained using the Helium-rebreathing technique [22]. Values were expressed as a percentage of the predicted value calculated using standardized reference equations [23]. Diffusing capacity of the lung for carbon monoxide (TLCO), expressed as [ml/(min*mmHg)], was measured according to current ATS/ERS guidelines [20]. It was obtained by a single-breath method using a dedicated gas-chromatography system (Biomedin, Padua, Italy). A 5-minute resting period was maintained before the measurement.

### Submaximal physical exercise

Participants performed the 6-minute walk test (6mWT) according to ATS guidelines [24]. The 6 mWT suits elderly patients [25], [26] and reliably assesses functional capacity. We chose to perform a submaximal exercise testing because, at variance form maximal exercise testing, it can be widely implemented in clinical settings. Moreover, a linear relationship between TLCO and workload has been demonstrated [27]. Walking distance was expressed as absolute value and as percent predicted using validated reference equations [28]. Dyspnoea was rated by the BORG scale [29].

### Sensors

The artificial olfactory system used for this study is the current version of the gas sensor array developed and fabricated at the University of Rome Tor Vergata. The array used for this study is composed of seven quartz microbalance (QMB) sensors covered with metalloporphyrins [30] as chemical interactive materials: Cu-TPP, Co-TPP, Zn-TPP, Mn-TPP, Fe-TPP, Sn-TPP, Ru-TPP are the seven metals selected. Sensors responses to VOCs result in seven frequency shifts of each of the QMB respect to their typical resonance frequency. This technology has been validated and its performance has been evaluated in gases and vapours calibration experiments for both single compounds and mixtures [1], [31], [32].

The sensor-VOCs interaction is mediated by weak bonds, such as Van der Waals, dipole-dipole, hydrogen and other ones depending upon the polarization status of VOCs. The variety of interaction mechanisms results in a variety of sensor responses, and this is the pre-condition for the data analysis treatment. [30].

Supplementary information, including a detailed list of validation studies, is available in Supporting Information S1.

### Breath sampling

Each subject had to fill a three liters tedlar bag (distributed by SKC, Eighty Four, PA, USA). The mouthpiece and the tubes used for the sampling apparatus were disposable; the valves were fabricated of Teflon, an inert and odorless material. Although alveolar gas volume has been shown to distinguish lung cancer from asthma patients better than the total exhaled breath [1], [33], [34], we collected the total exhaled breath because in a proof of concept study it seemed logical to expand the source of information.

### Statistical method

A total number of 25 individuals have been considered for data analysis. Thus, the final dataset is composed of a total number of 74 measurements, 3 weekly repetitions for 24 of the probands and two for one of them.

Data collected by the electronic nose consist of a set of 7-dimensional patterns, one for each of the 3 measurements performed on each of the 25 patients. Thus the final dataset is an array, which can be explored either by a mono-dimensional analysis (sensor by sensor) or by a multidimensional one. Both analyses aim to test reproducibility.

All the elaborations have been performed in MATLAB (The MathWorks Inc. Natick, Massachusetts, U.S.A.) environment. For Multivariate Data Analysis the PLS-Toolbox (Eigenvector Research, Inc. Wenatchee, USA) has been used.

Data are described as means (and 95% confidence intervals, CI) for continuous variables, and as percentages for categorical variables.

Mono-dimensional data analysis: to verify the reproducibility, the median, the confidence interval and the outliers have been calculated and represented in a single plot (boxplot) for each patient, together with the parameters registered by respiratory function tests. This plot allows a visual comparison of e-nose and respiratory function data. Moreover the two standard deviations have been normalized to the mean value for VOCs and respiratory function parameters in each patient. We remind that, in the absence of standard reference values, each subject qualifies as the best reference for him/herself. This makes standard deviation the most convenient statistical figure.

The Spearman rho has also been calculated as a measure of correlation between VOC patterns derived by selected sensors and individual respiratory function and clinical parameters.

A Partial Least Square Discriminant Analysis (PLS-DA) has been performed on the 7-dimensional data array to built a model able to predict the respiratory function parameters. The PLS-DA model has been cross-validated via the leave-one-out criterion and, then, the Root Mean Square Error (in Cross-Validation) (RMSECV) of the model in the prediction of the respiratory function indexes has been computed. The (RMSECV) provides a measure of the robustness of the PLS-DA model [35].

## Results

Mean age of all participants was 73.6 years, 64% were males.

Anthropometric, health status and clinical data of control and COPD groups are summarized in table 1. Body weight and smokers distribution did not show significant differences amongst COPD groups; as expected, former smokers were more prevalent in late stage COPD. The two stage IV GOLD patients who were never smokers reported exposure industrial dust and kitchen vapours, respectively. The lowest values of 6 minute walked distance and the highest dyspnoea score were recorded in GOLD stage 4 and 3. Prevalence of comorbidites did not distinguish COPD groups except for a higher prevalence of obstructive sleep apnoea in GOLD 4 stage(p = 0.034). No cases of chronic heart failure, liver or kidney diseases, nor peptic ulcer or any other related gastric disease were recorded. Drug use increased for increasing GOLD stage, and only GOLD 4 patients were on long term Oxygen therapy. The mobility subscore of the Barthel's index also worsened with increasing GOLD stage, whereas the dependency subscore, which reflects proficiency in basic ADLs, did not distinguish groups. The frequency of exacerbations was maximal in GOLD 4 group.

**Table 1:** Demographic and clinical characteristics of control subjects and COPD patients grouped according to GOLD stage of disease severity.

	Controls (N = 5)	GOLD1 (N = 5)	GOLD2 (N = 5)	GOLD 3 (N = 5)	GOLD4 (N = 5)	P
**Age; mean (SD)**	73.9 (3.8)	74.4 (8.9)	75.6 (3.2)	78.8 (4.5)	73.2 (6.3)	0.128
**Males, n (%)**	2 (40)	3 (60)	4 (80)	5 (100)	3 (60)	0.439
**BMI; mean (SD)**	25.1 (4.2)	23.6 (4.4)	28.0 (3.5)	26.2 (3.9)	29.8 (3.8)	0.186
**Smoking status, n (%)**						0.081
**Current**	0 (0)	1 (20)	0 (0)	2 (40)	1 (20)	
**Former**	1 (20)	2 (40)	5 (100)	3 (60)	2 (40)	
**Never**	4 (80)	2 (40)	0 (0)	0 (0)	2 (40)	
**Pack/Year; mean (SD)**	5.0 (11.2)	23.0 (22.2)	55.5 (33.0)	49.0 (17.5)	41.2 (56.6)	0.099
**MRC dyspnea score; mean** **(SD)**	0.40 (0.9)	1.4 (1.3)	1.0 (1.2)	3.0 (0.2)	2.2 (0.8)	0.004
**6 MWD in meters; mean** **(SD)**	569 (97)	387 (61)	323 (54)	323 (147)	307 (96)	0.004
**Diabetes mellitus, n (%)**	1 (20)	1 (20)	2 (40)	2 (40)	0 (0)	0.381
**Ischemic heart disease (%)**	0 (0)	1 (20)	1 (20)	2 (40)	1 (20)	0.711
**Pulmonary hypertension/cor pulmonare, n (%)**	0 (0)	1 (20)	0 (0)	1 (20)	1 (20)	0.684
**Bronchiectasis, n (%)**	0 (0)	0 (0)	0 (0)	1 (20)	0 (0)	0.439
**Obstructive sleep apnoea,** **n (%)**	0 (0)	0 (0)	0 (0)	0 (0)	2 (40)	0.034
**Number of inhaled** **medications; mean (SD)**	0	2.0 (0.7)	2.2 (1.3)	2.8 (0.4)	3.0 (1.2)	0.005
**Oxygen therapy, n (%)**	0 (0)	0 (0)	0 (0)	0 (0)	5 (100)	<0.001
**Barthel's total Score; mean** **(SD)**	102 (0)	98.6 (2.3)	93.4 (11.1)	85.0 (7.3)	84.0 (7.6)	0.001
**Barthel's selfcare subscore; mean (SD)**	51.0 (0)	49.6 (2.2)	47.8 (5.6)	47.2 (5.0)	46.6 (4.9)	0.450
**Barthel's mobility subscore; mean (SD)**	51.0 (0)	49.0 (2.7)	45.0 (6.2)	37.8 (2.9)	37.6 (3.8)	<0.001
**Number of acute** **exacerbation of COPD in the past year;** **mean (SD)**	0	0.8 (0.8)	1.0 (1.4)	2.6 (1.7)	3.0 (0.7)	0.02
**White blood cell count** **× 10^3^ ; mean (SD)**	5.27 (0.69)	7.91 (1.30)	7.55 (2.11)	8.46 (1.82)	7.32 (1.77)	0.052
**Erytrocyte sedimentation** **rate; mean (SD)**	12.7 (18.2)	32.8 (13.8)	22.2 (8.4)	40.0 (45.2)	28.6 (13.6)	0.355
**C-reactive protein; mean (SD)**	1.6 (0.3)	2.1 (2.7)	1.95 (2.1)	7.82 (12.8)	3.93 (2.5)	0.395

Comparisons between groups were performed by χ-square test for categorical variables, and one-way ANOVA analyses (followed by Bonferroni post-hoc multiple comparison adjustment) for continuous variables.

Respiratory function data are available in Table S1.

### Reproducibility and consistency analysis

**Figure 1:**
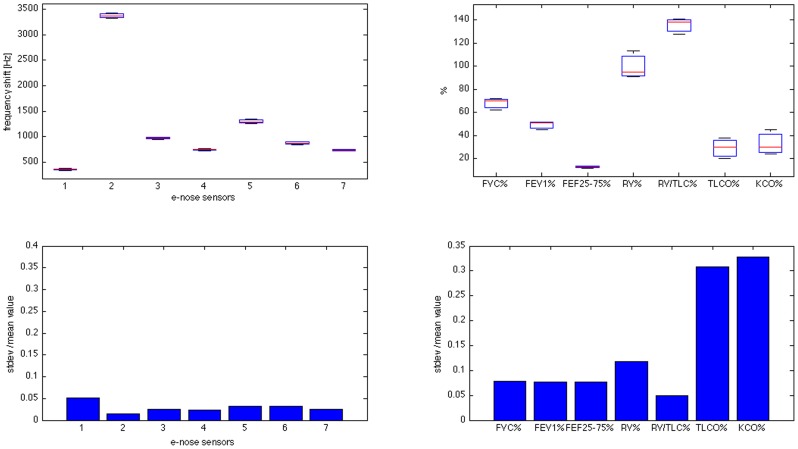
Boxplots and bar-graphs comparing e-nose data and respiratory function indexes in terms of reproducibility in a control individual. Boxplot and standard deviation normalized to the mean value respectively; First columns: boxplots and normalized standard deviations for the six e-nose sensor responses. Second column: boxplots and normalized standard deviations for six selected respiratory function indexes (% of FVC, FEV1, FEF25–75, RV, RV/TLC, TLCO, KCO).

**Figure 2:**
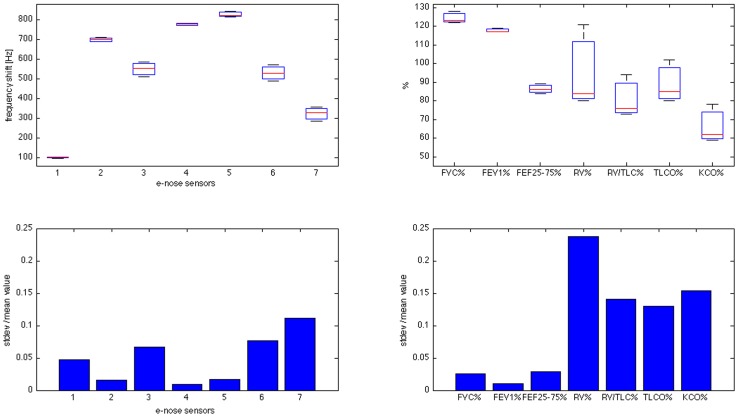
Boxplots and bar-graphs comparing e-nose data and respiratory function indexes in terms of reproducibility in a GOLD 4 patient. Boxplot and standard deviation normalized to the mean value respectively. First columns: boxplots and normalized standard deviations for the six e-nose sensor responses. Second column: boxplots and normalized standard deviations for six selected respiratory function indexes (% of FVC, FEV1, FEF25–75, RV, RV/TLC, TLCO, KCO).

Reproducibility of measures obtained by gas sensor array and that of pulmonary function tests are comparatively showed in figures 1 and 2, respectively. The figures are composed by two panels for each individual, the upper one providing boxplots of intra observer variability for individual e nose sensors (left upper panel) and for main respiratory function indexes (right upper panel). The amplitudes of the standard deviation for every measured parameter are reported in the lower panel. Figure 1 refers to a control individual (lines first and second), figure 2 to a GOLD4 patient. Reproducibility of VOCs fingerprints was extremely fair in both control and GOLD 4 groups. In GOLD 1–3 groups, VOCs fingerprints were much less reproducible than respiratory function tests. Figures show the worst performance in terms of reproducibility and repeatability observed in the two extreme groups and have therefore only an explanatory function. Complete data on all patients are available in the online supplementary files (Figures S1–S25).

The coefficients of correlation between the sensor patterns and the main respiratory function indexes are reported in Table 2. The correlation was highly significant for most respiratory function parameters with respect to five of the seven sensors composing the array. All the parameters reported are the ones with p values <0.001. The greatest Spearman's rhos are registered for the FEV1% with respect to the five sensors, the lowest rhos for RV/TLC and for KCO%. The Cu-Buti-TPP sensor showed the strongest correlations with all the respiratory function parameters.

**Table 2:** Coefficients of correlation between the sensor patterns and the main respiratory function indexes (only rho with p<0.001).

sensors	Cu-Buti-TPP	Co-Buti-TPP	Zn-Buti-TPP	Sn-Buti-TPP	Ru-Buti-TPP
**FVC %**	−0.60	−0.56	−0.67	−0.58	−0.67
**FEV1 %**	−0.81	−0.63	−0.79	−0.66	−0.79
**FEV1/VC**	−0.76	−0.57	−0.70	−0.56	−0.70
**FEF25–75%**	−0.76	−0.51	−0.70	−0.57	−0.70
**PEF %**	−0.69	−0.52	−0.63	−0.48	−0.63
**MEF75%**	−0.80	−0.54	−0.71	−0.56	−0.70
**MEF50%**	−0.78	−0.52	−0.72	−0.57	−0.71
**MEF25%**	−0.52	−0.36	−0.45	−0.45	−0.48
**RV/TLC**	0.48	0.49	0.54	0.41	0.53
**FRC/TLC**	0.70	0.47	0.57	0.47	0.58
**TLCO(Va)%**	−0.63	−0.39	−0.51	−0.41	−0.47
**KCO%**	−0.57	−	−0.41	−	−0.38

A simpler representation of e-nose breath-print correlation with the respiratory function parameters is given by a multivariate approach: the whole array data has been used to built a PLS-DA model to predict the parameters reported in table 2. The model gave very promising Root Mean Square Error in Cross Validation (RMSECV), as reported in table 3.

**Table 3:** Root Mean Square Error (in Cross-Validation) (RMSECV) for the Partial Least Square – Discriminant Analysis (PLS-DA) model for the respiratory function parameters based on the e-nose data.

Parameter	RMSECV	Predicted-real Spearman rho
FVC %	16.282	0.72
FEV1 %	14.273	0.87
FEV1/FVC	9.956	0.77
FEF 25–75%	15.83	0.85
PEF %	16.76	0.78
MEF 75%	17.082	0.87
MEF 50%	14.435	0.90
MEF 25%	30.071	0.60
RV/TLC	9.584	0.56
FRC/TLC	7.79	0.73
TLCO (Va) %	21.852	0.67
KCO%	19.076	0.63

The RMSECV provides a measure of how reliably PLS-DA model predicts respiratory function indexes.

## Discussion

This study proves that VOC pattern is fairly reproducible within healthy subjects and hypoxemic COPD patients and, in the COPD population, correlates with expiratory flows and, to a lesser extent, with indexes of air trapping and DLCO, but not with RV and TLC.

The fair intra-individual reproducibility of VOC guarantees for the quality of e-nose based research. The only previous report pertained to repeatability of two measurements within 5 minutes [4]. Our observation refers to three measurements at weekly intervals and, thus, definitively proves that VOC patterns are highly reproducible, though less reproducible than spirometric parameters. However, most of spirometric parameters reflect flows and volumes which are expected to vary negligibly provided that the respiratory manoeuvre is correctly performed. On the other hand, VOC fingerprint is a sort of multidimensional index of exhaled air composition [35]. Even in highly repeatable conditions, the composition of the exhaled air fluctuates in the presence of bronchial inflammation. Indeed, a large diurnal variability in exhaled hydrogen peroxide levels, a marker of oxidative stress, has been reported in stable COPD [36]. Thus, VOCs reflect a more complex and dynamically changing process than spirometry does.

The fact that reproducibility of VOCs pattern was better in GOLD 4 than in GOLD 1–3 COPD patients deserves some comment. Given that hypoxemia was the hallmark of GOLD 4 stage, it is likely that these patients had a sort of distinctive and, thus, highly reproducible hypoxemic VOCs pattern [37], [38]. Contrasting this hypothesis is the fact that hypoxemic patients were on continuous Oxygen therapy, and Oxygen supplementation is expected to prevent the hypoxemic damage. However, in real life these patients variably experience discontinuous hypoxemia because the FiO2 is usually tailored to at rest and not to nocturnal and effort needs [39]. On the other hand, non hypoxemic COPD, corresponding to 1–3 GOLD stages, likely are a more heterogeneous population. Indeed, COPD is currently considered an umbrella definition embracing a cluster of variably related diseases [40]. For instance, the recently proposed “frequent exacerbator” phenotype might harbour an important inflammatory status in the bronchial tree and, thus, be exposed to fluctuations in VOCs pattern [41]. Thus, VOCs patterns of COPD patients prior to the onset of hypoxemia likely are a complex reality worthy of study by a qualitative analysis of breath which is out of the realm of e nose technology.

VOC correlated very strictly with MEF 50 and MEF 75 (for three of the seven sensors), which refer to air exhaled from small calibre airways, the site of important inflammation in COPD. [42]. Small airways have also been reported to contribute meaningfully to bronchial obstruction and to correlate with patient centred outcomes in COPD [43]. FEV1 also significantly correlates with VOC for three of the seven sensors), suggesting that a well defined link exists between bronchial obstruction and pattern of exhaled breath. Conceivably, both flows, which are decreased by bronchial obstruction, and VOC reflect disease severity and, to some extent, disease type, i. e. COPD phenotype. The same is not true of RV and TLC, two indexes useful to characterize the emphysematous version of COPD: they were completely unrelated to VOC as if bronchial and not parenchymal damage contributed to determine the exhaled breath pattern. It is also of interest that two strong correlates of VOC, MEF50 and MEF75, are commonly considered “minor” respiratory function indexes mainly because of their great inter-individual variability [43]. Indeed, FEV1 and not MEF50 or MEF75 is used to rate COPD severity. Finally, the correlation between WBC blood count and VOC points at bronchial inflammation as a potential determinant of VOCs pattern. Thus, carefully defining the phenotype of COPD and rating bronchial inflammation are expected to improve our knowledge of VOCs determinants and clinical meaning with regard to present findings.

This study has some limitations. First, we had no direct measures of bronchial inflammation such as NO concentration in the exhaled breath. Second, we recorded VOCs fingerprints, but we could not identify their components by dedicated analyses. Third, we had no counter-check of whether the recorded VOC pattern is typical of stable COPD as we did not measure VOCs in exacerbated COPD. Fourth, 4 out of our 20 COPD patients were still smokers, and this might have contributed to VOC patterning. However, differences in bronchial inflammation between smokers with and without COPD have been reported to be merely quantitative [44]. Finally, we cannot exclude that the highly reproducible VOCs pattern in GOLD 4 patients simply reflect hypoxemia and, then, might recur in non COPD conditions characterized by chronic hypoxemia.

This study also has important strengths. Indeed, it provides the first demonstration of VOC reproducibility in both normal and COPD subjects. Furthermore, no other study has so far comparatively tested respiratory function, biological and health status correlates of VOC in COPD. Thus, our data pave the way to the assessment of VOC classificatory and discriminatory properties in clinical practice. Indeed, due to the good reproducibility and the important correlation with several respiratory function indexes, VOC pattern might qualify as a surrogate measure of disease severity or else as a diagnostic alternative to spirometry for the large fraction of people unable to meet the quality standards of spirometry [8]. Dedicated research is well founded on our findings.

In conclusion, this study proves that VOCs are fairly reproducible within healthy and hypoxemic COPD subjects, less among non hypoxemic COPD patients, and are strictly related to spirometric measures of expiratory flows. Research is needed to verify to which extent VOCs patterns change during exacerbations and whether distinctive VOC patterns point at selected COPD phenotypes.

## Supporting Information

Figure S1 **Gold2.** Boxplots of all the 25 individuals. Figures relative to the complete data set on all patients. Each figure (S1–S25), like in figure 1 in the text. The figure is composed by two panels for each individual, the upper one providing boxplots of intra observer variability for individual e nose sensors (left upper panel) and for main respiratory function indexes (right upper panel). The amplitudes of the standard deviation for every measured parameter are reported in the lower panel. All the figures are listed in alphabetical order (referred to the names of the patients), just to indicate the Gold standard classification.
(PNG)

Figure S2 **Gold1.**
(PNG)

Figure S3 **Gold2.**
(PNG)

Figure S4 **Gold1.**
(PNG)

Figure S5 **Gold2.**
(PNG)

Figure S6 **Control.**
(PNG)

Figure S7 **Gold2.**
(PNG)

Figure S8 **Gold3.**
(PNG)

Figure S9 **Gold1.**
(PNG)

Figure S10 **Gold4.**
(PNG)

Figure S11 **Control.**
(PNG)

Figure S12 **Gold3.**
(PNG)

Figure S13 **Gold3.**
(PNG)

Figure S14 **Gold1.**
(PNG)

Figure S15 **Gold 2.**
(PNG)

Figure S16 **Control.**
(PNG)

Figure S17 **Gold3.**
(PNG)

Figure S18 **Gold1.**
(PNG)

Figure S19 **Gold4.**
(PNG)

Figure S20 **Control.**
(PNG)

Figure S21 **Control.**
(PNG)

Figure S22 **Gold4.**
(PNG)

Figure S23 **Gold4.**
(PNG)

Figure S24 **Gold3.**
(PNG)

Figure S25 **Gold4.**
(PNG)

Supporting Information S1 **E-nose technical specifications file.**
(DOCX)

Table S1 **Respiratory function tests of control subjects.and COPD patients grouped according to GOLD stage of disease severity.**
(DOC)
