# Supplementary figures and images for: Reproducibility and Respiratory Function Correlates of Exhaled Breath Fingerprint in Chronic Obstructive Pulmonary Disease
Source: PLoS One. 2012 Oct 15;7(10):e45396. doi: 10.1371/journal.pone.0045396 (PMC3471938; doi:10.1371/journal.pone.0045396)

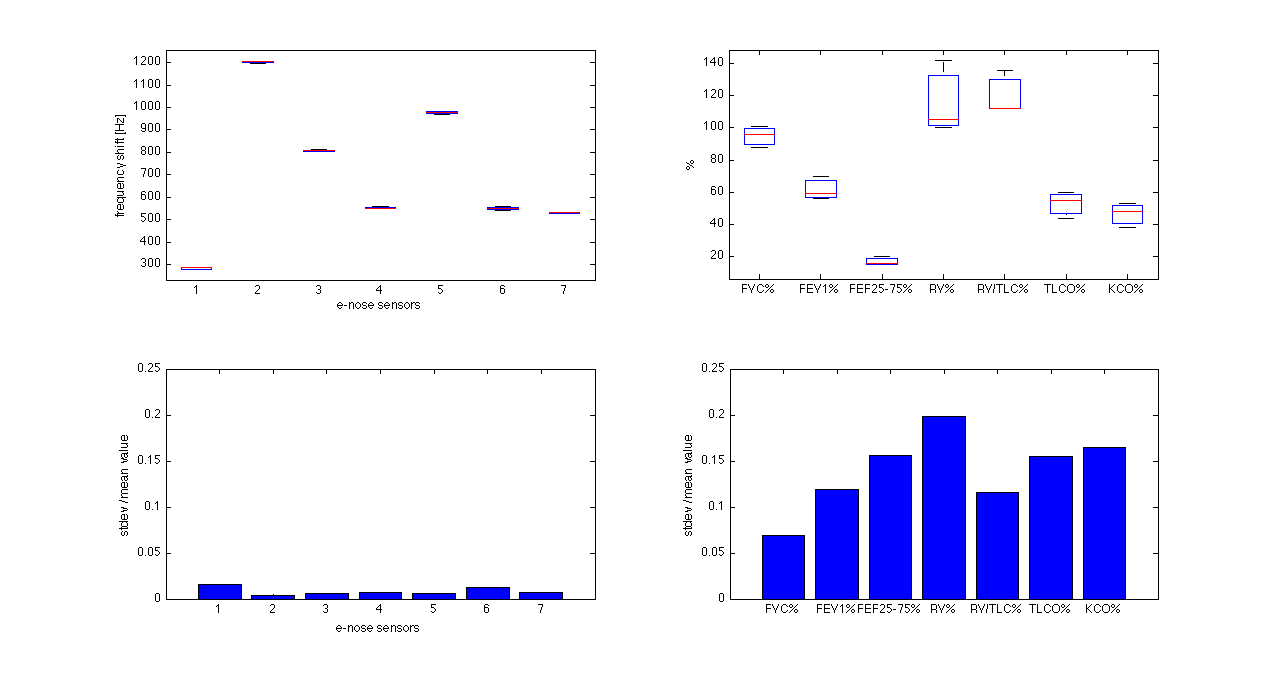

Supplement: Figure S1 — Gold2. Boxplots of all the 25 individuals. Figures relative to the complete data set on all patients. Each figure (S1–S25), like in figure 1 in the text. The figure is composed by two panels for each individual, the upper one providing boxplots of intra observer variability for individual e nose sensors (left upper panel) and for main respiratory function indexes (right upper panel). The amplitudes of the standard deviation for every measured parameter are reported in the lower panel. All the figures are listed in alphabetical order (referred to the names of the patients), just to indicate the Gold standard classification. (PNG) [file pone.0045396.s001.png]

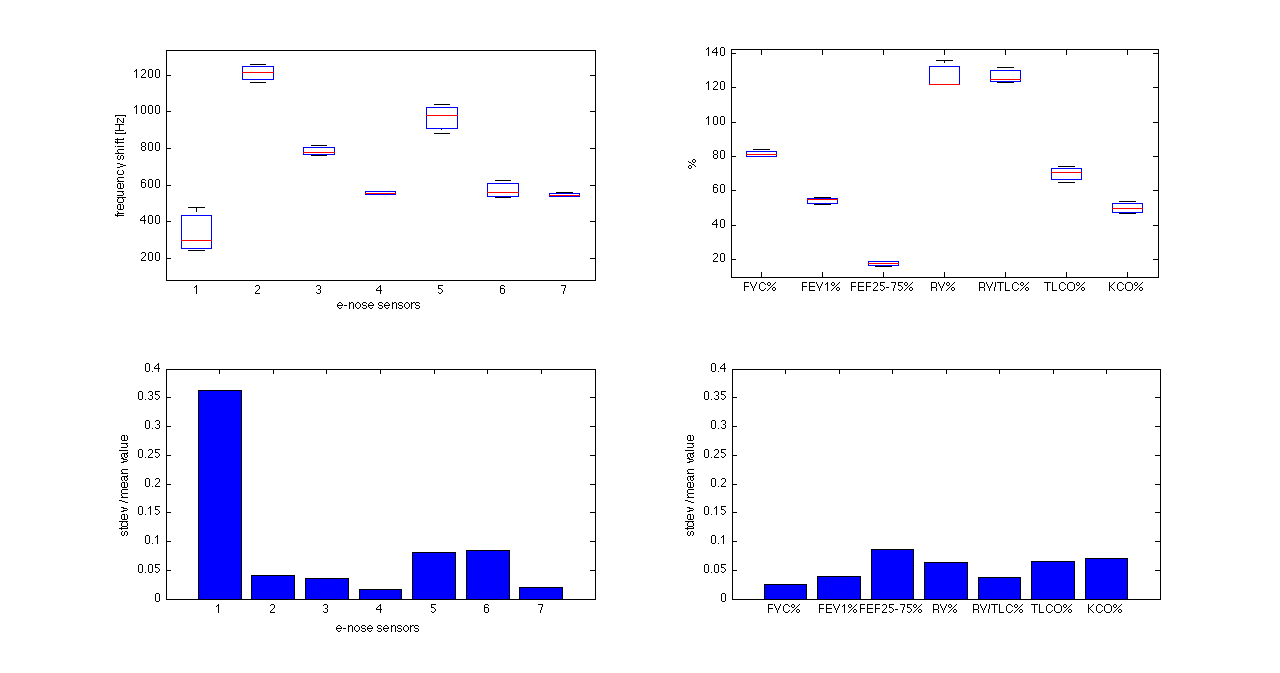

Supplement: Figure S2 — Gold1. (PNG) [file pone.0045396.s002.png]

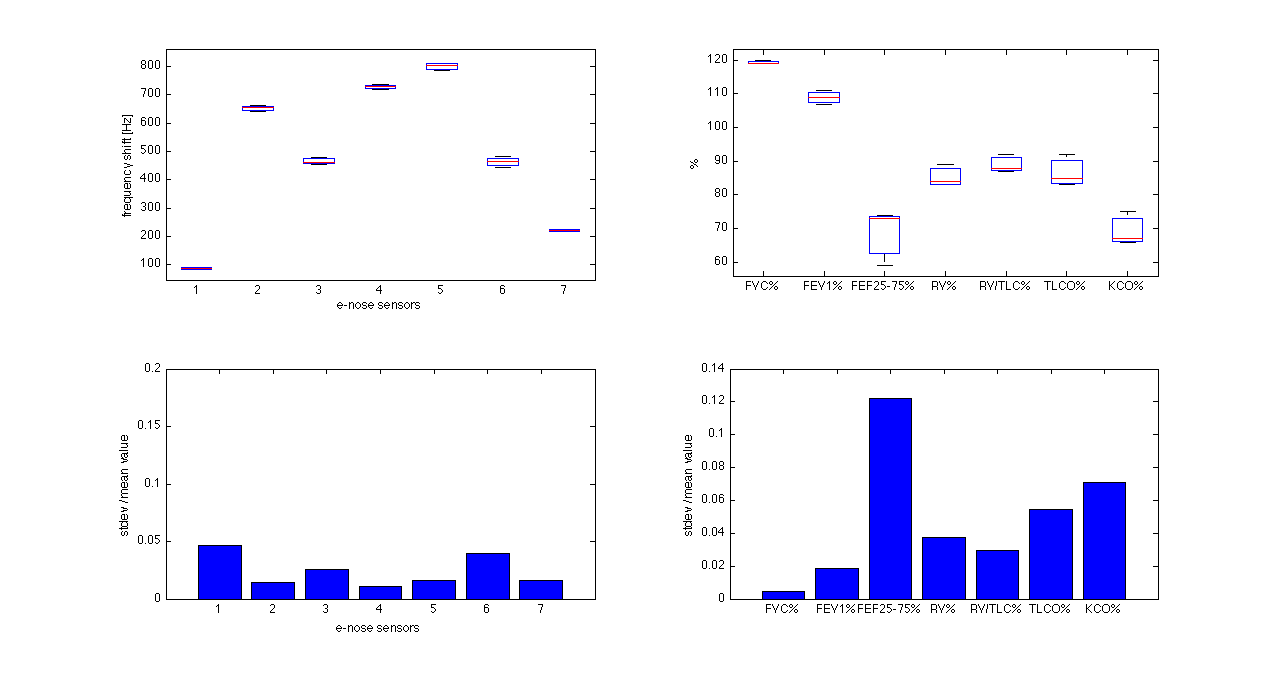

Supplement: Figure S3 — Gold2. (PNG) [file pone.0045396.s003.png]

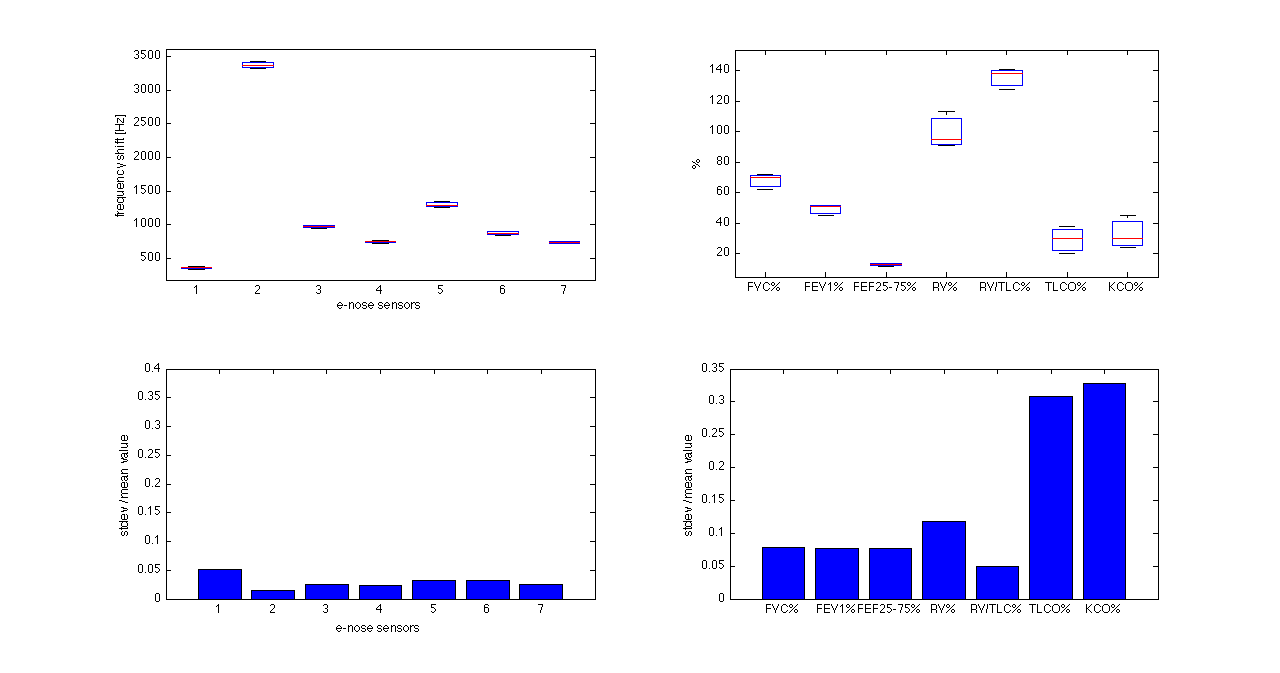

Supplement: Figure S4 — Gold1. (PNG) [file pone.0045396.s004.png]

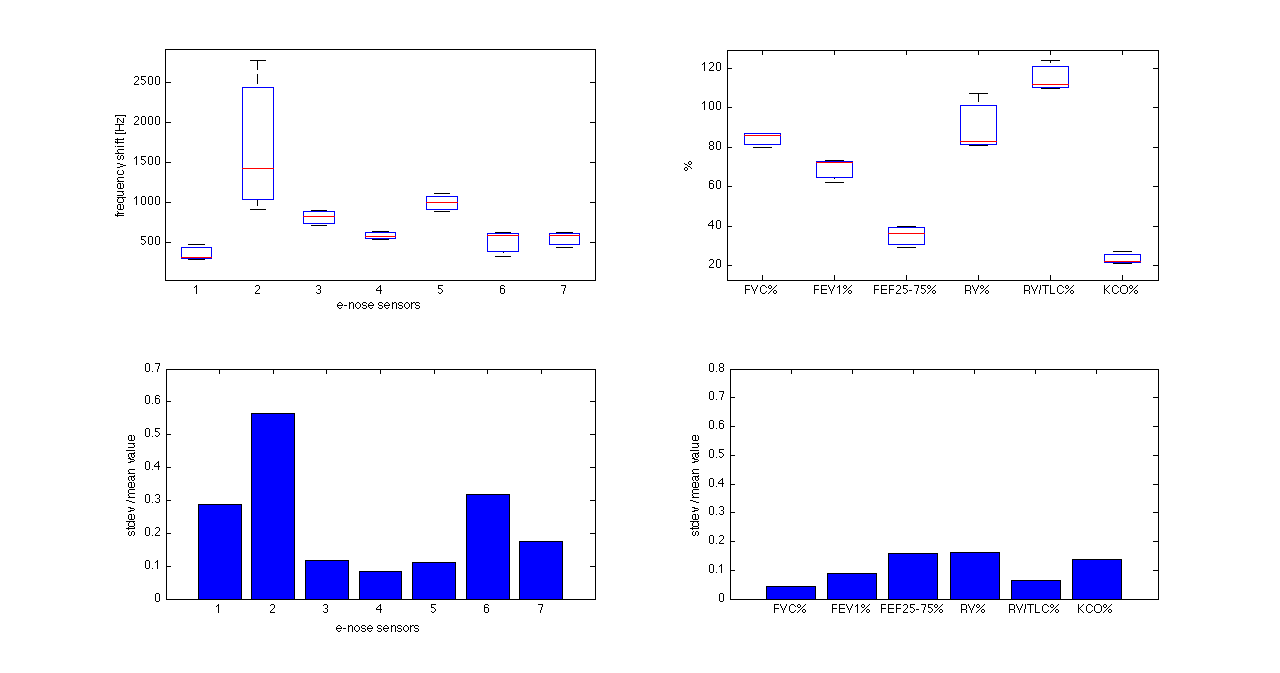

Supplement: Figure S5 — Gold2. (PNG) [file pone.0045396.s005.png]

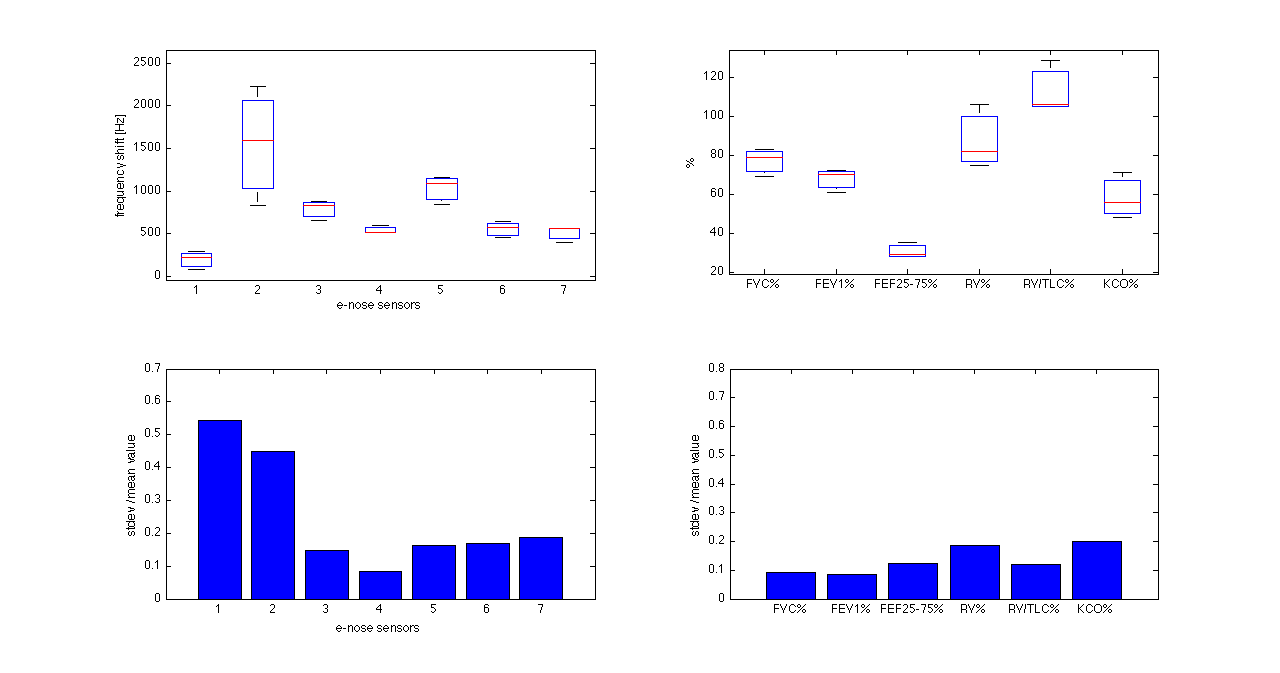

Supplement: Figure S6 — Control. (PNG) [file pone.0045396.s006.png]

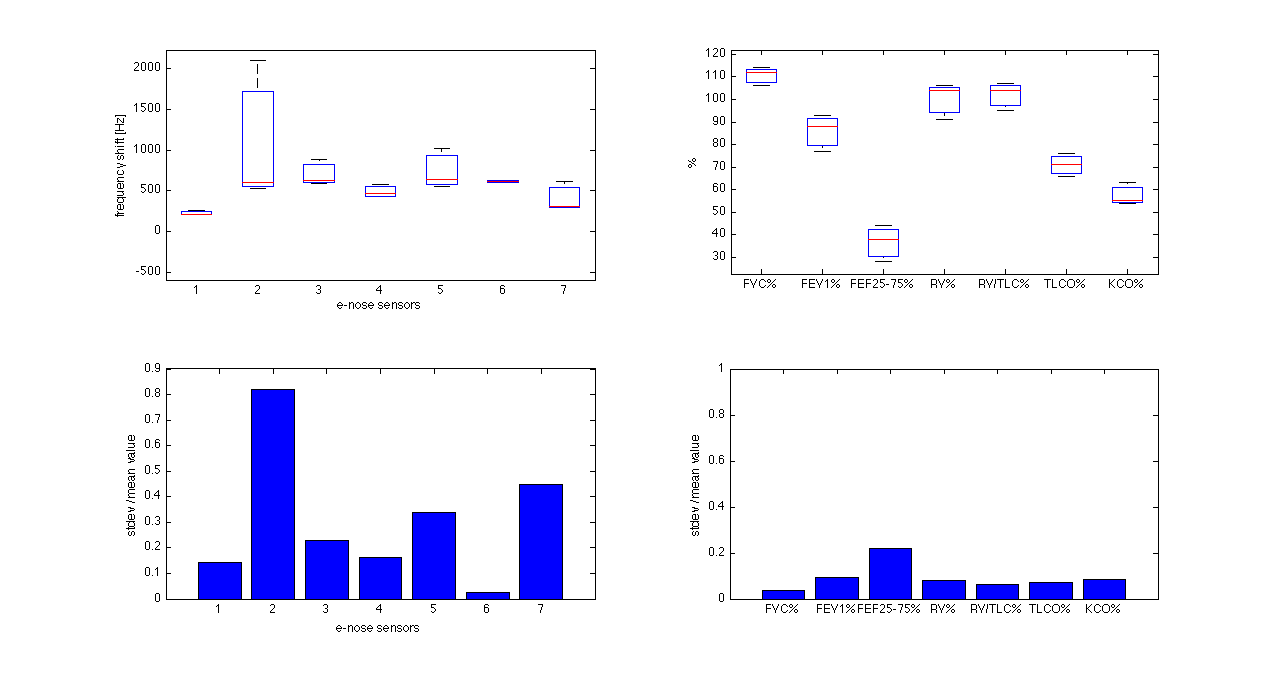

Supplement: Figure S7 — Gold2. (PNG) [file pone.0045396.s007.png]

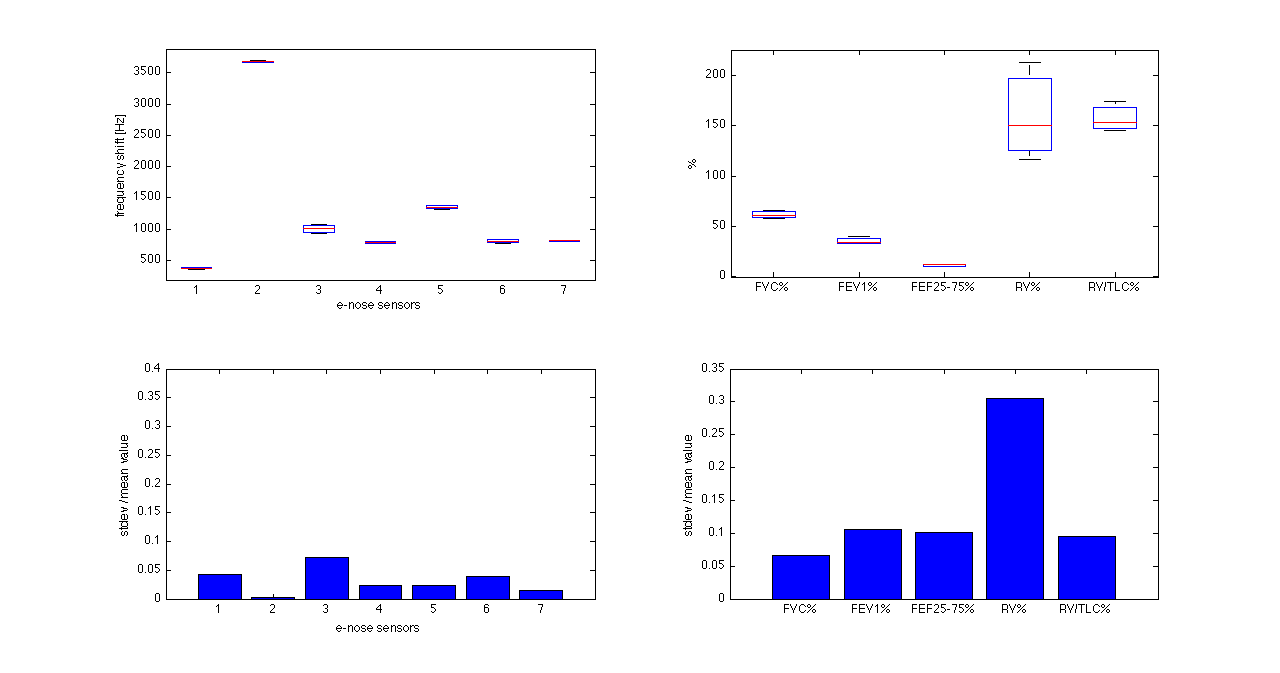

Supplement: Figure S8 — Gold3. (PNG) [file pone.0045396.s008.png]

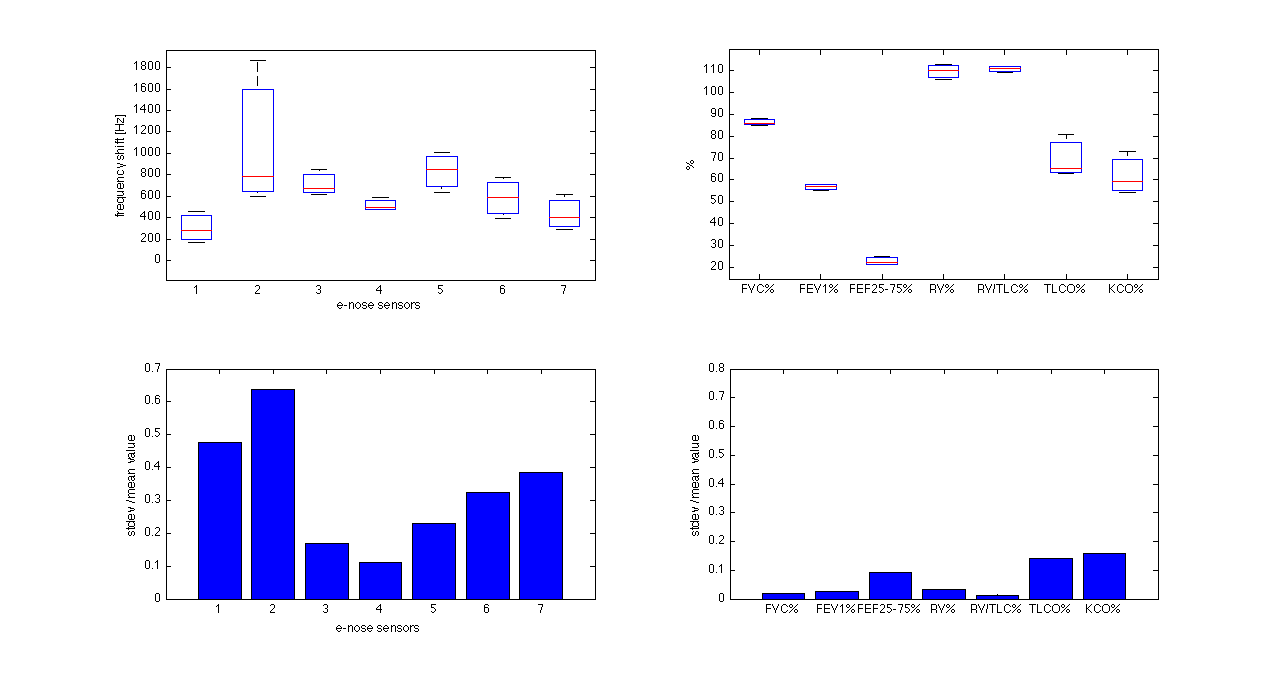

Supplement: Figure S9 — Gold1. (PNG) [file pone.0045396.s009.png]

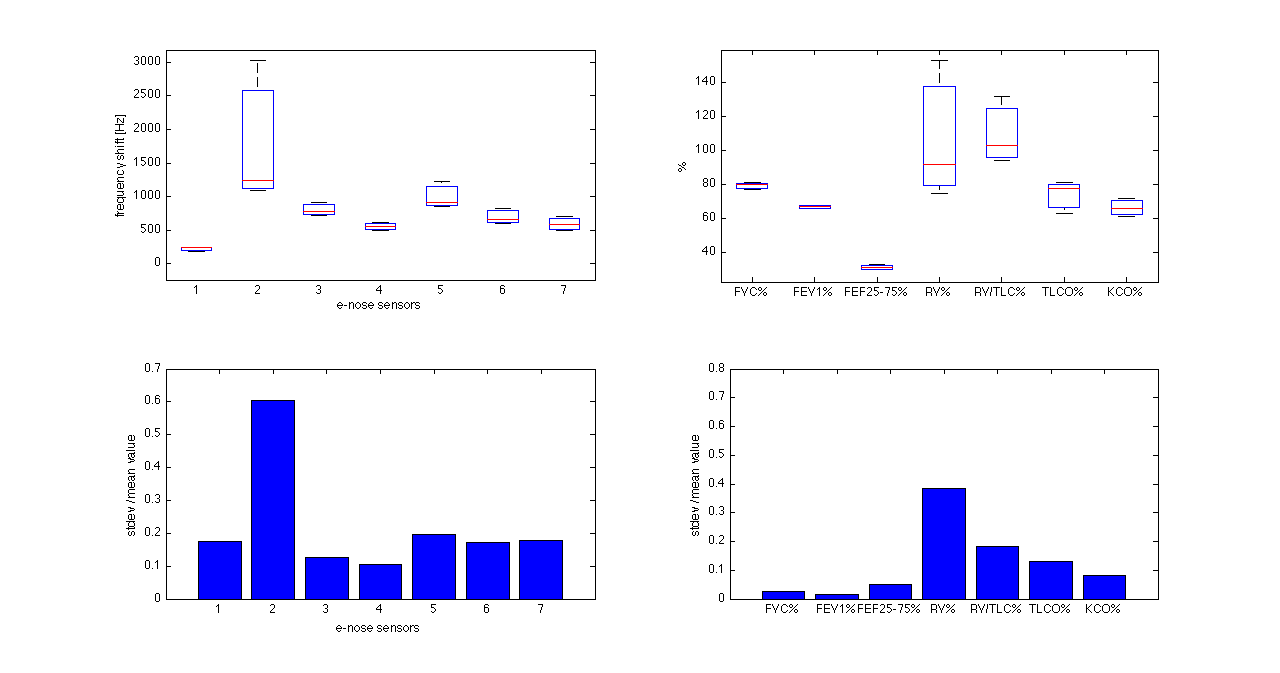

Supplement: Figure S10 — Gold4. (PNG) [file pone.0045396.s010.png]

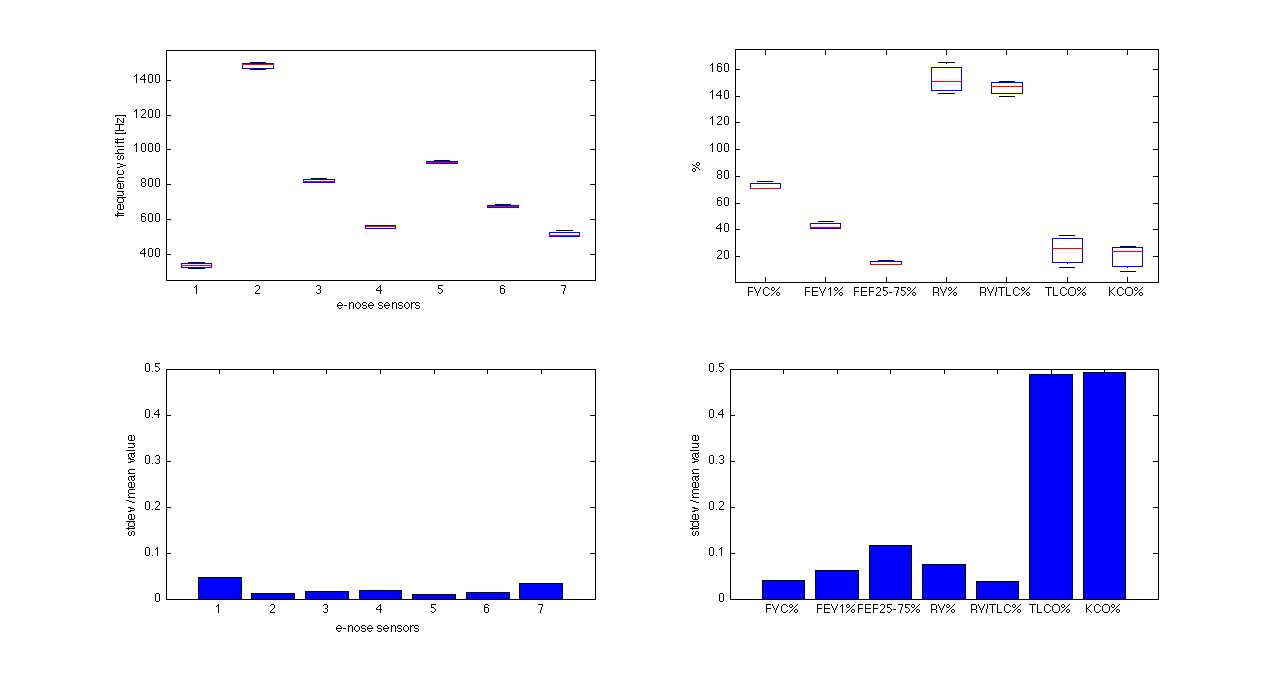

Supplement: Figure S11 — Control. (PNG) [file pone.0045396.s011.png]

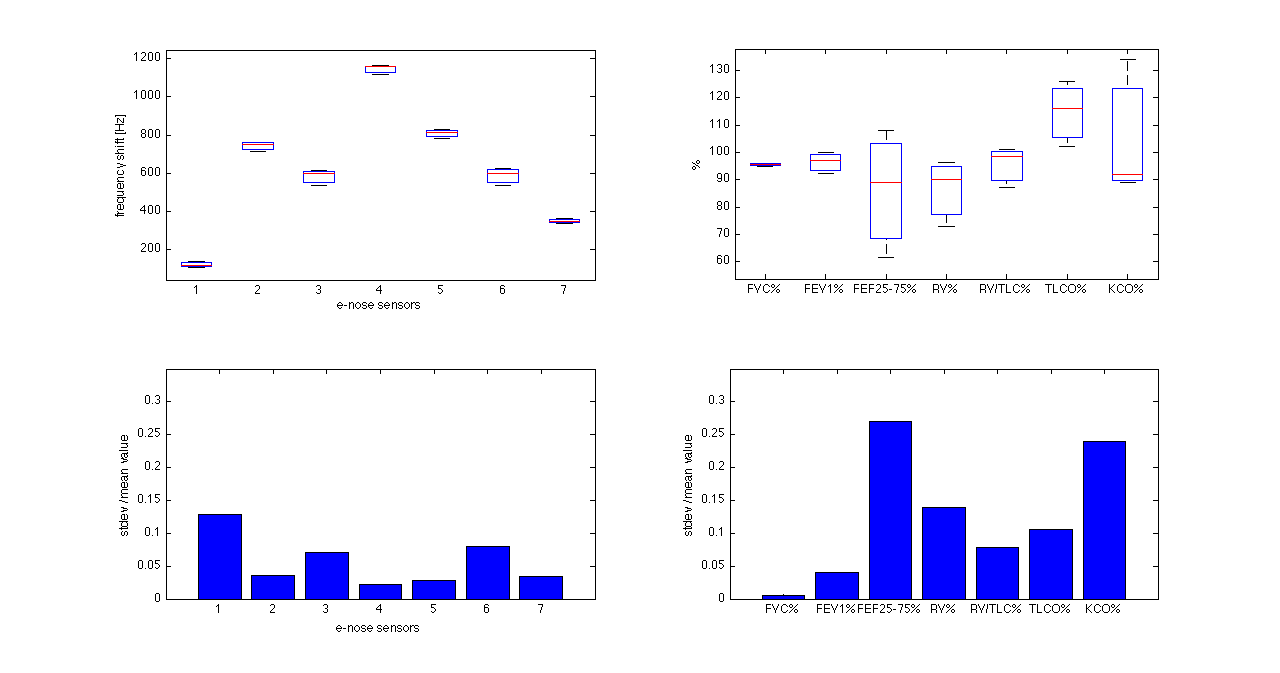

Supplement: Figure S12 — Gold3. (PNG) [file pone.0045396.s012.png]

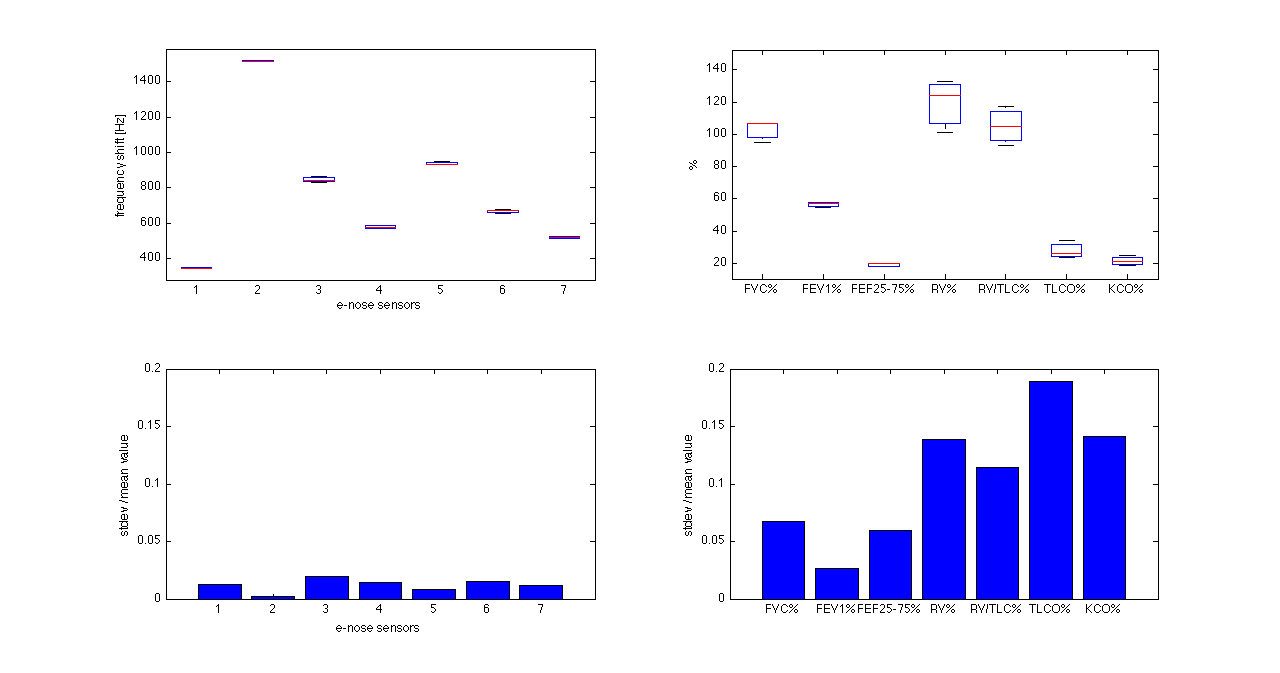

Supplement: Figure S13 — Gold3. (PNG) [file pone.0045396.s013.png]

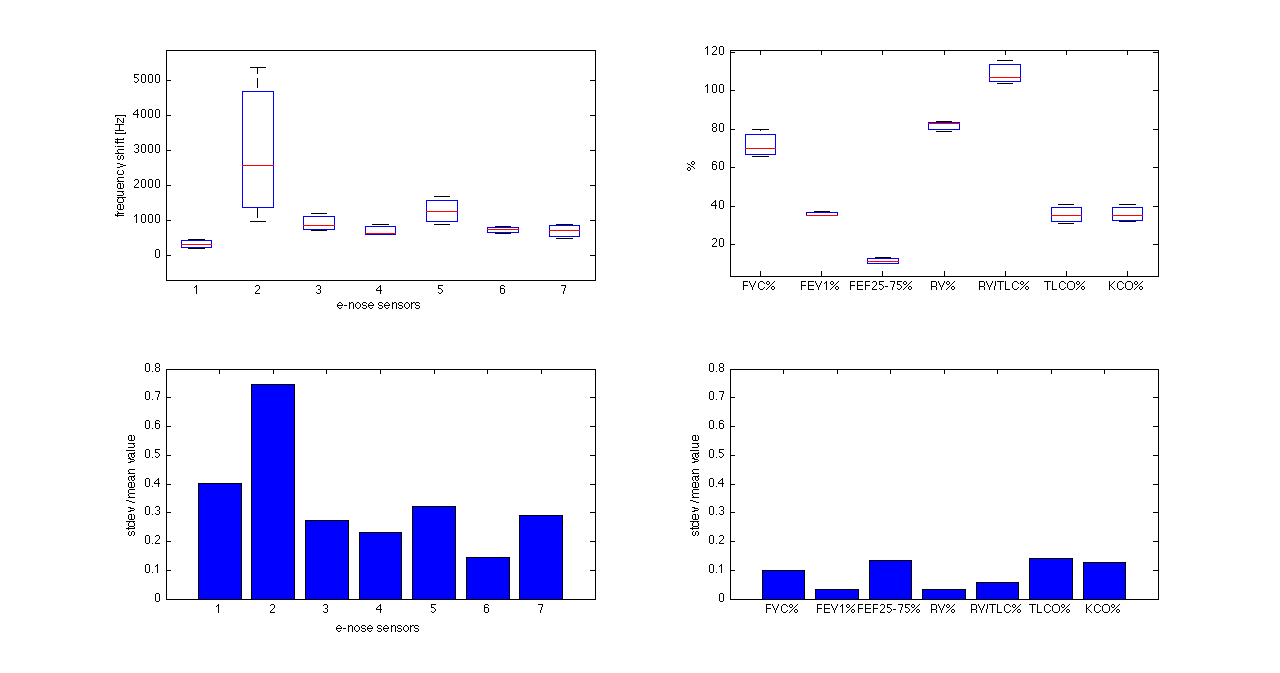

Supplement: Figure S14 — Gold1. (PNG) [file pone.0045396.s014.png]

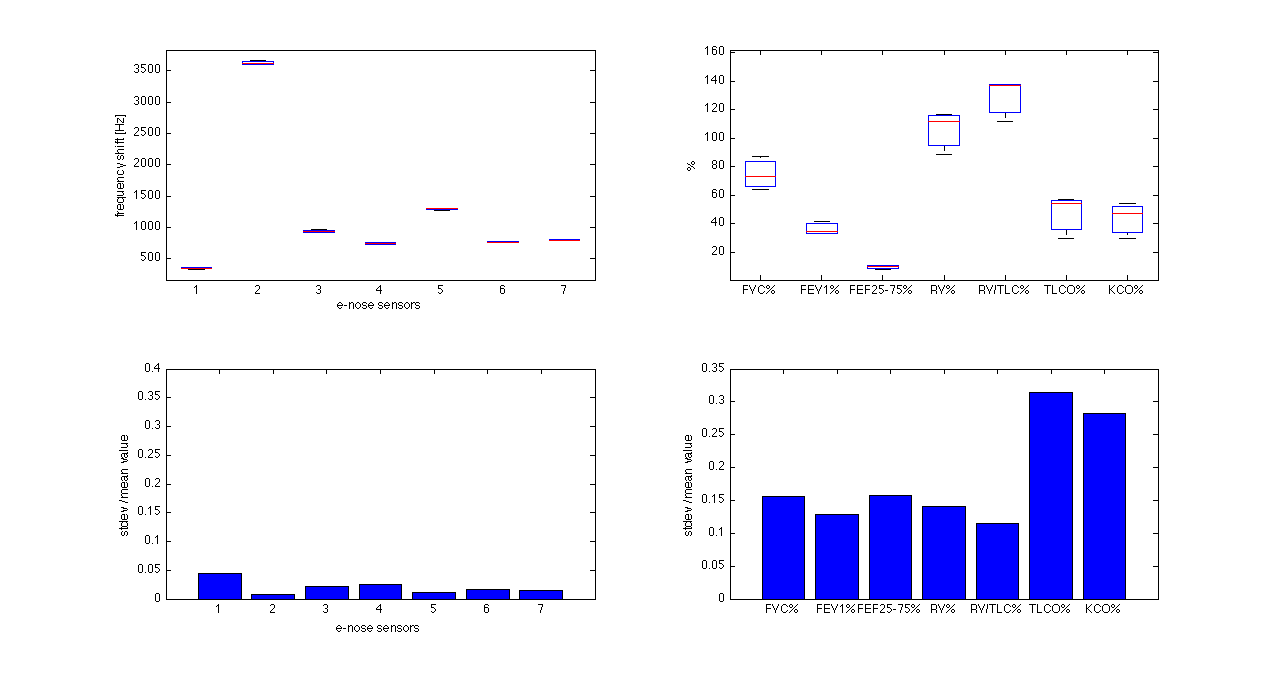

Supplement: Figure S15 — Gold 2. (PNG) [file pone.0045396.s015.png]

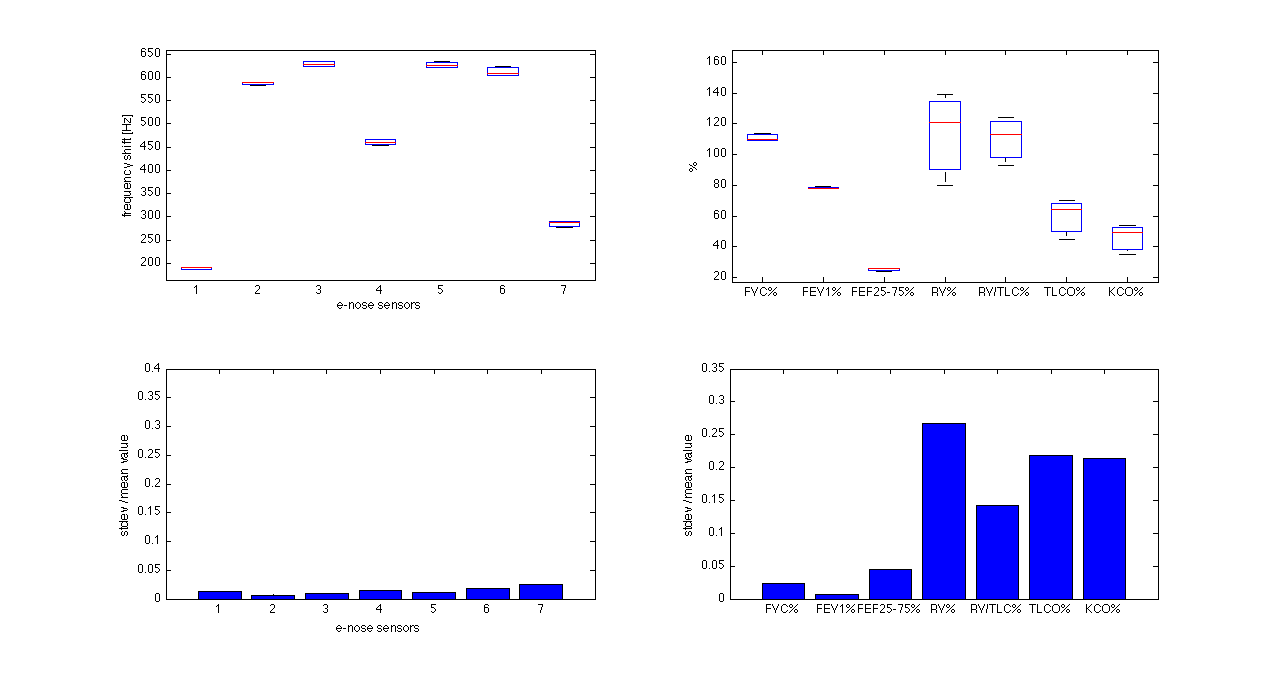

Supplement: Figure S16 — Control. (PNG) [file pone.0045396.s016.png]

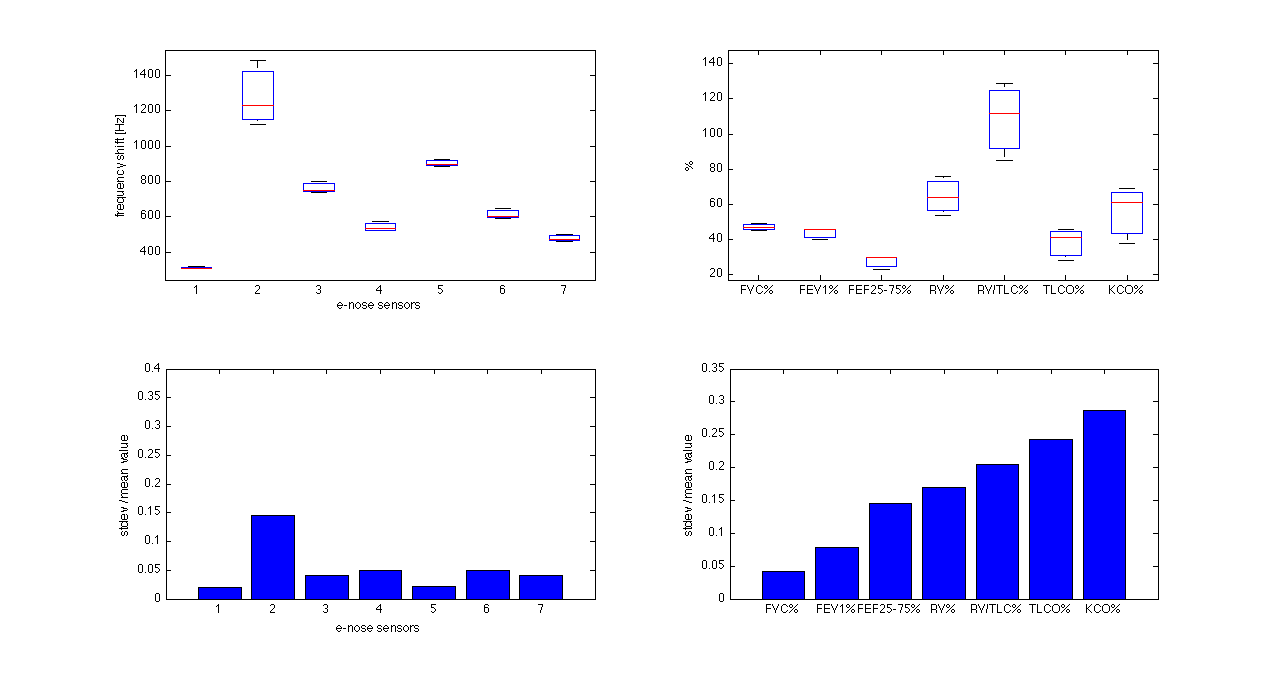

Supplement: Figure S17 — Gold3. (PNG) [file pone.0045396.s017.png]

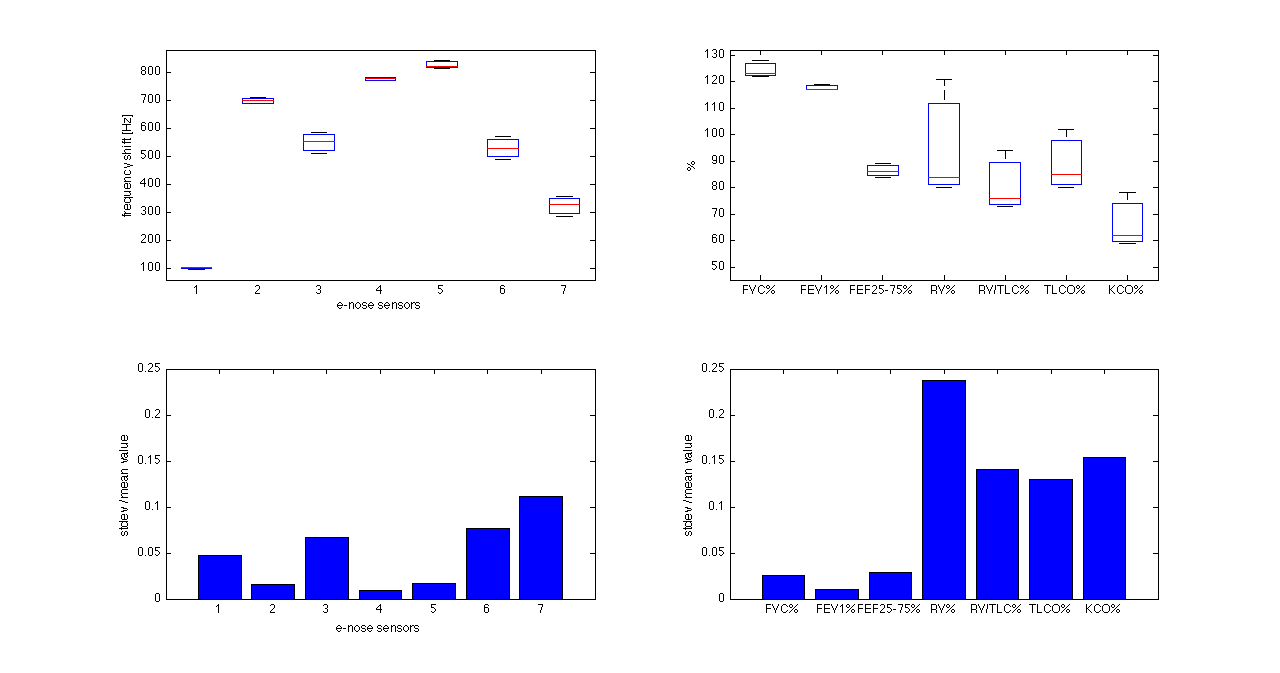

Supplement: Figure S18 — Gold1. (PNG) [file pone.0045396.s018.png]

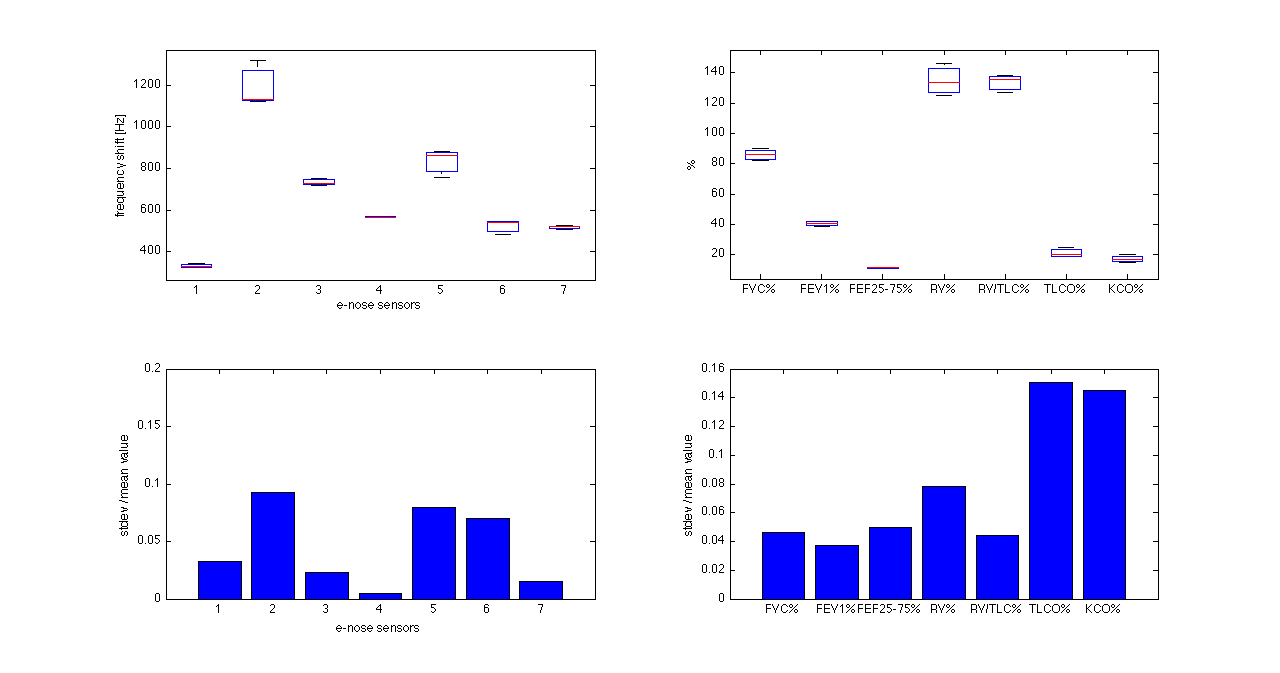

Supplement: Figure S19 — Gold4. (PNG) [file pone.0045396.s019.png]

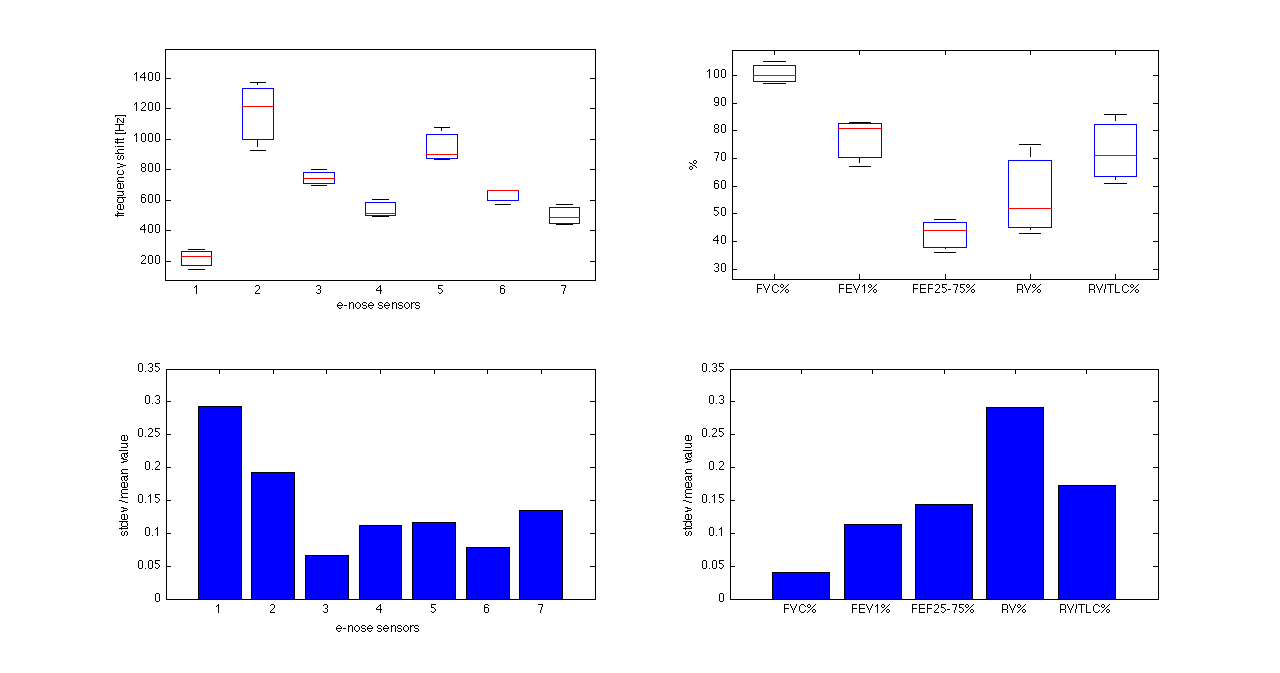

Supplement: Figure S20 — Control. (PNG) [file pone.0045396.s020.png]

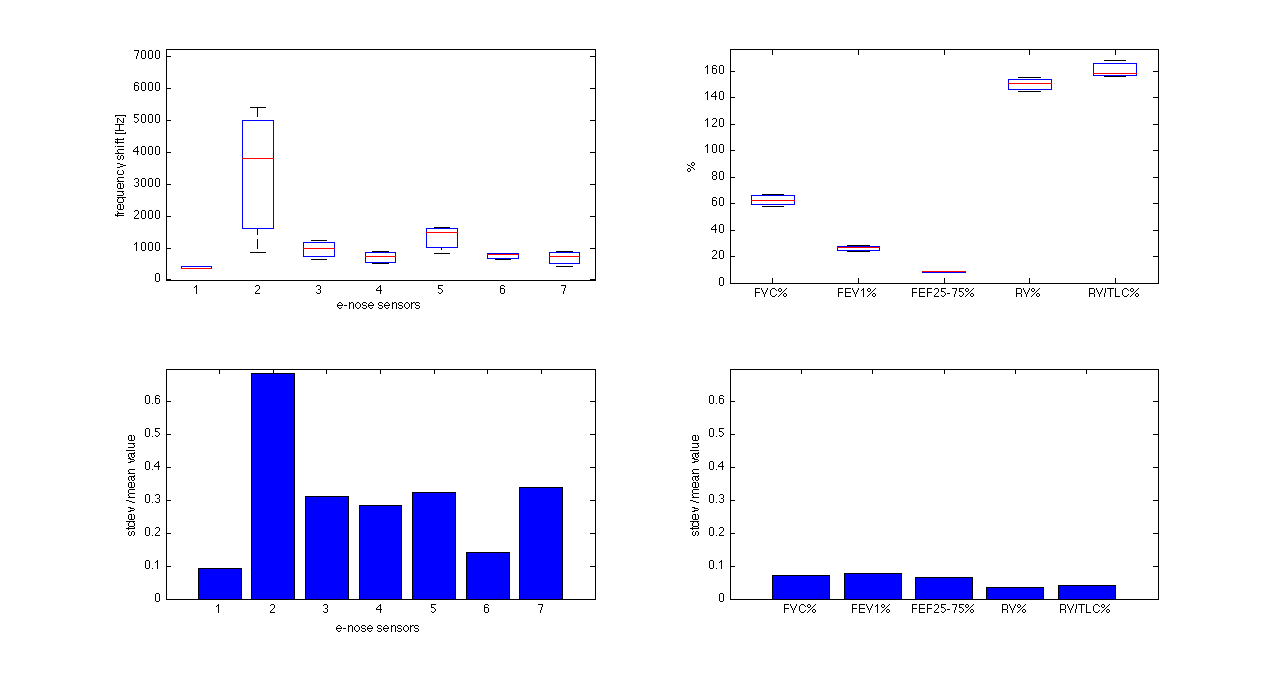

Supplement: Figure S21 — Control. (PNG) [file pone.0045396.s021.png]

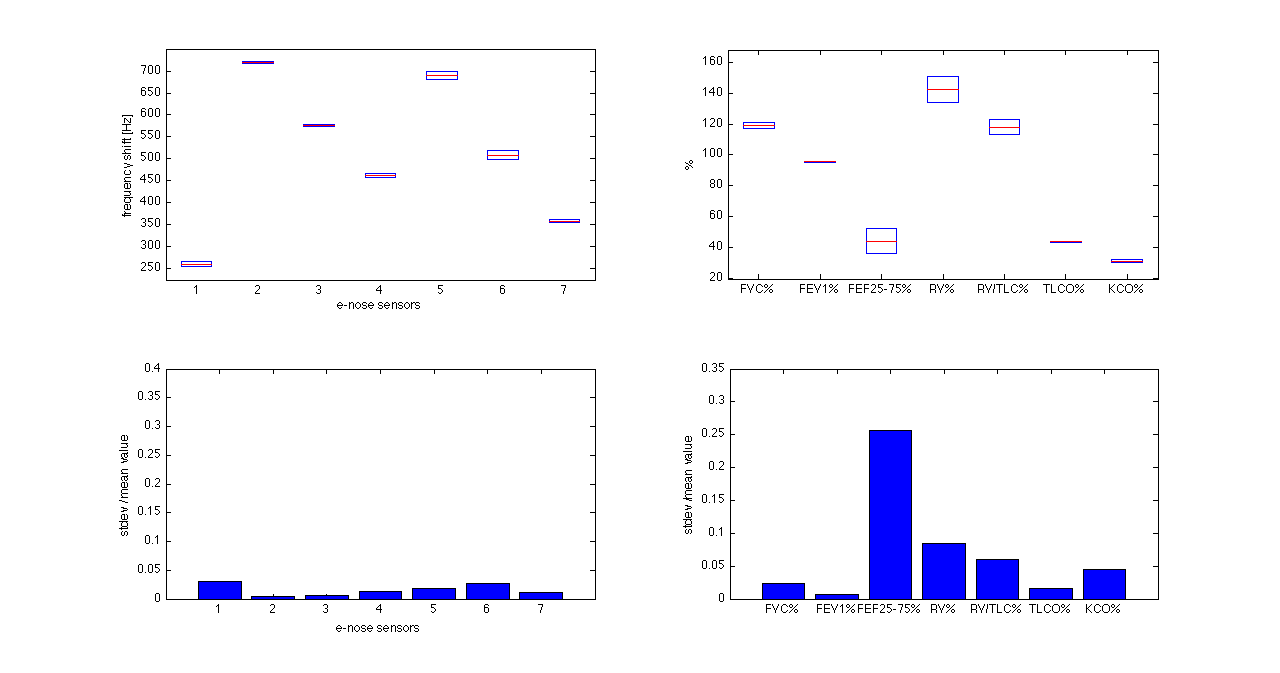

Supplement: Figure S22 — Gold4. (PNG) [file pone.0045396.s022.png]

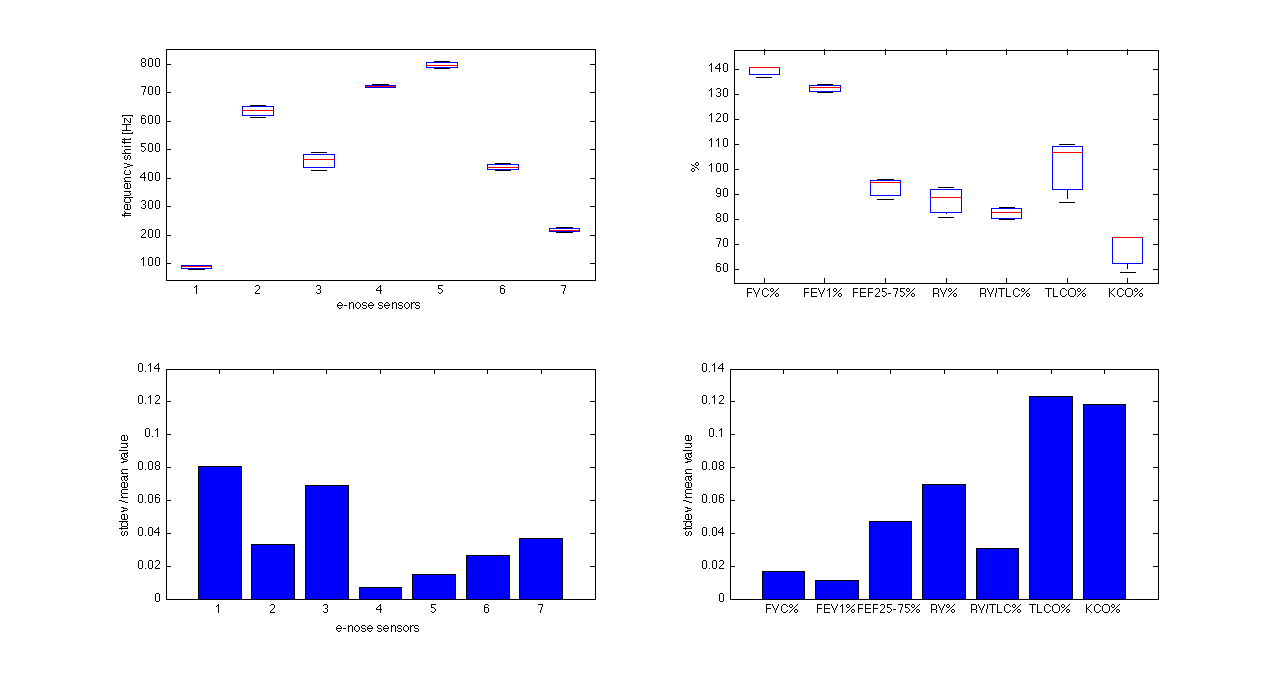

Supplement: Figure S23 — Gold4. (PNG) [file pone.0045396.s023.png]

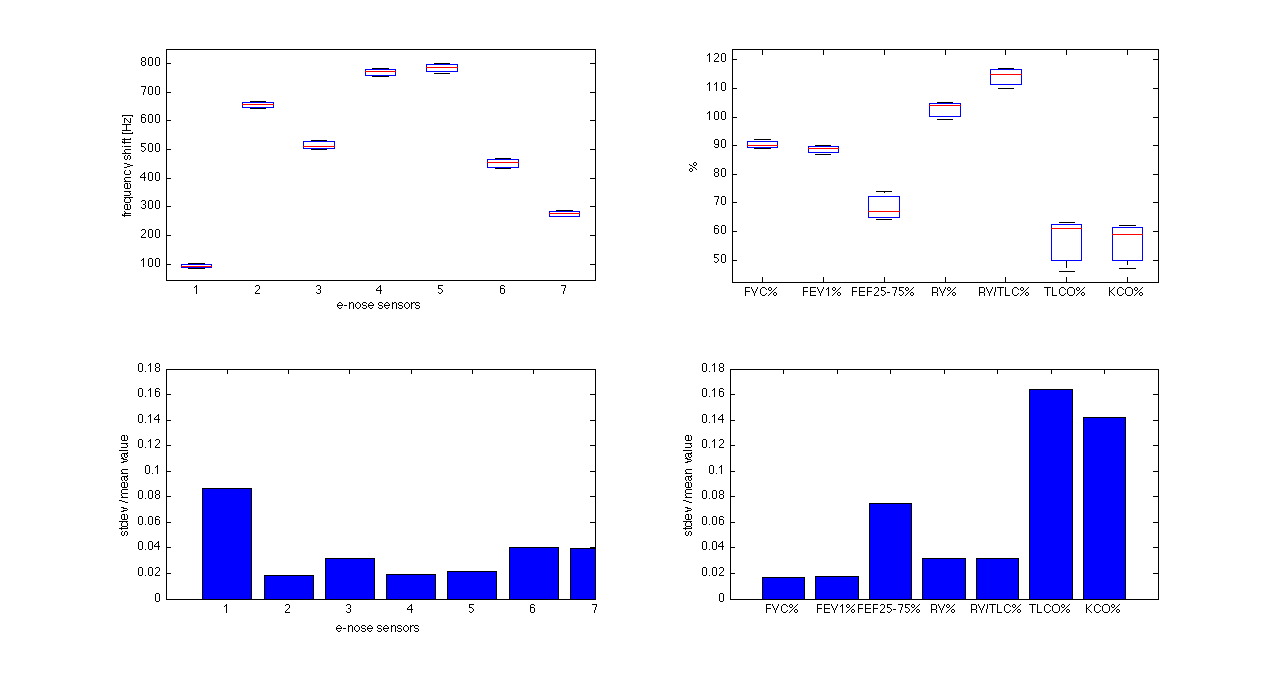

Supplement: Figure S24 — Gold3. (PNG) [file pone.0045396.s024.png]

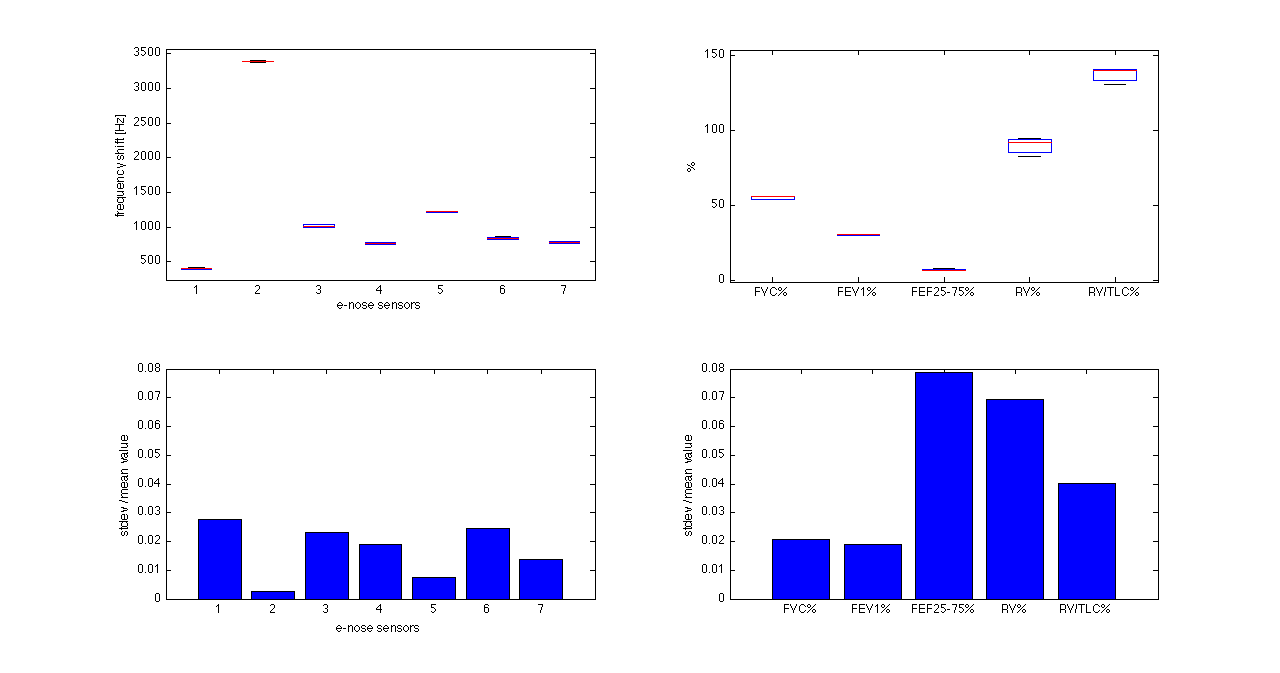

Supplement: Figure S25 — Gold4. (PNG) [file pone.0045396.s025.png]
