# Supplementary material for: Reproducibility and Respiratory Function Correlates of Exhaled Breath Fingerprint in Chronic Obstructive Pulmonary Disease
Source: PLoS One. 2012 Oct 15;7(10):e45396. doi: 10.1371/journal.pone.0045396 (PMC3471938; doi:10.1371/journal.pone.0045396)
Supplement: Supporting Information S1 — E-nose technical specifications file. (DOCX) [file pone.0045396.s026.docx]

**Supporting Information S1**

**E-nose technical specifications**

A gas sensor array in which the chemical interactive material is non selective is intended to imitate the natural olfaction mechanisms, whose olfactive receptors are non selective. This is the reason why these devices are dubbed electronic noses [1].

The electronic nose used in this work is based on Quartz microbalances. These sensors are based on a piezoelectric crystals whose resonance frequency is directly proportional to the mass load graving on its surface. This relationship was ruled by Sauerbrey [2] as reported below:

$$\Delta f_{0}=-\cdot\frac{2f_{^{0}}^{2}}{A\sqrt{\mu_{q}\rho_{q}}}\Delta m_{s}$$

where A is the coated area, ρ_q_ the quartz density, μ_q_ is the shear stiffness, f_0_ is the fundamental frequency.

The chemical interactive material used to cover the six QMB crystals are six different metalloporphyrins. Each metallo-porphyrin is characterized by six different metals (reported in the text) coordinated at the center of the pyrrole [3].

The features extracted by the sensors responses consists of the frequency shifts registered between two values: the reference value obtained by a nitrogen flow and the measure value given by the equilibrium between adsorbed and desorbed volatile molecules.

The six-dmensional data sets are analyzed by multivariate data analysis techinques, such as Principal Component Analysis (PCA) and Partial Least Square Discriminant Analysis (PLS-DA) [4].

References

[1] Persaud, K. & Dodd, G., Analysis of discrimination mechanisms in the mammalian olfactory system using a model nose. Nature 299, 352–355 (1982).

[2] G. Sauerbrey (1959), Use of quartz crystal vibrator for weighing thin films on a microbalance, Zitung Physik.

[3] Di Natale C., Paolesse R., D’Amico A., Metalloporphyrins based artificial olfactory receptors. Sens Actuators B 2007;121:238-46.

[4] Simon M.S., David J., Zulfiqur A. Data Analysis for electronic nose systems. Microchim Acta 156,183-207 (2007).
