# Supplementary material for: Reproducibility and Respiratory Function Correlates of Exhaled Breath Fingerprint in Chronic Obstructive Pulmonary Disease
Source: PLoS One. 2012 Oct 15;7(10):e45396. doi: 10.1371/journal.pone.0045396 (PMC3471938; doi:10.1371/journal.pone.0045396)
Supplement: Table S1 — Respiratory function tests of control subjects.and COPD patients grouped according to GOLD stage of disease severity. (DOC) [file pone.0045396.s027.doc]

**Table S1**

Respiratory function tests of control subjects and COPD patients grouped according to GOLD stage of disease severity.

|  | Controls **(N=5)** | GOLD1 **(N=5)** | GOLD 2 **(N=5)** | GOLD 3 **(N=5)** | GOLD 4 **(N=5)** | **p-value** |
| --- | --- | --- | --- | --- | --- | --- |
| **FEV1 obs.** | 2.20 (0.37) | 1.62 (0.26) | 1.50 (0.34) | 1.16 (0.37) | 0.70 (0.08) | <0.001 |
| **FEV1 (% predicted)** | 106.7 (14.4) | 80.14 (10.18) | 61.5 (6.66) | 44.0 (7.44) | 35.73 (8.5) | <0.001 |
| **VC obs.** | 2.81 (0.44) | 2.76 (0.34) | 2.63 (0.53) | 2.48 (0.88) | 1.69 (0.37) | <0.001 |
| **VC  (% predicted)** | 105.6  (17.5) | 99.86  (11.3) | 83.13  (5.78) | 75.20 (17.0) | 62.73 (8.63) | <0.001 |
| **FEV1/FVC  (%)** | 78.1  (3.9) | 61.0  (5.0) | 54.8  (5.8) | 44.3 (10.5) | 43.1 (9.5) | <0.001 |
| **TLC obs** | 4.63 (0.57) | 4.81 (0.52) | 5.50 (0.99) | 5.57 (1.33) | 4.50 (1.27) | 0.009 |
| **TLC  (% predicted)** | 91.5  (11.5) | 94.0  (16.6) | 89.9  (9.2) | 87.6 (21.0) | 85.6 (16.6) | 0.628 |
| **RV obs.** | 1.80 (0.19) | 2.15 (0.51) | 2.76 (0.62) | 2.89 (0.90) | 2.80 (1.04) | <0.001 |
| **RV  (% predicted)** | 84.0 (10.1) | 97.6  (31.7) | 109.3  (23.2) | 110.7 (35.2) | 121.2 (35.5) | 0.01 |
| **DlCO  (Hb-COHb)** | 17.3  (7.6) | 15.4  (4.1) | 10.5  (4.1) | 6.9 (2.5) | 5.3 (3.2) | <0.001 |
| **DlCO  (Hb-COHb)  (% predicted)** | 81.4  (29.8) | 66.3  (10.1) | 52.2  (20.1) | 29.6 (9.3) | 28.4 (17.4) | <0.001 |
| **KCO** | 3.6  (1.1) | 2.81  (0.4) | 2.3  (0.9) | 1.45 (0.8) | 1.54 (0.7) | <0.001 |
| **KCO  (% predicted)** | 67.9  (26.2) | 56.7  (10.3) | 40.2  (15.1) | 30.3 (16.7) | 29.4 (15.0) | <0.001 |
